# Divergent innate and adaptive immune trajectories after bariatric surgery in people with severe obesity

**DOI:** 10.3389/fimmu.2026.1951003

**Published:** 2026-10-29

**Authors:** Jule Gawor, Andrea Deinzer, Maria Wick, Christian Krautz, Inaya Hayek, Christian Schwartz

**Affiliations:** 1 Mikrobiologisches Institut - Klinische Mikrobiologie, Immunologie und Hygiene, Universitätsklinikum Erlangen and Friedrich-Alexander-Universität (FAU) Erlangen-Nürnberg, Erlangen, Germany; 2 Chirurgische Klinik, Universitätsklinikum Erlangen and Friedrich-Alexander-Universität (FAU) Erlangen-Nürnberg, Erlangen, Germany; 3 FAU Immunomedicine (FAU I-MED), Erlangen, Germany

**Keywords:** bariatric surgery, eosinophils, macrophage polarization, obesity, type 2 immunity

## Abstract

**Background:**

Obesity disrupts type 2 immune cell populations in white adipose tissue, replacing the homeostatic network of group 2 innate lymphoid cells (ILC2s), eosinophils, T helper 2 (Th2) cells, and alternatively activated macrophages (AAMs) with pro-inflammatory type 1 populations. Whether this remodelling reflects permanent immune impairment or a reversible shift in cellular equilibrium, and to what extent bariatric surgery restores type 2 immunity, remain incompletely understood.

**Methods:**

We analysed selected type 2 immune cell populations in visceral white adipose tissue (WAT) and peripheral blood from persons with severe obesity (people with obesity, PWO) scheduled for or having undergone bariatric surgery (sleeve gastrectomy, gastric bypass), as well as non-obese controls. Using flow cytometry, quantitative PCR, and *in vitro* polarization assays, we assessed immune cell frequencies, transcription factor expression, cytokine profiles, and functional polarization capacity across lean, pre-operative, and post-operative states.

**Results:**

Obesity was associated with decreased eosinophil and CD8^+^ T cells frequencies in WAT, accompanied by an increase in CD4^+^ frequency and a shift from Th2 toward Th1 predominance, as well as elevated PD-1 expression on T cell subsets. PWO after bariatric surgery showed reduced CD8^+^ T cell frequencies and increased CD4^+^ T cells compared to lean controls. Macrophage polarization capacity was increased in PWO after surgery compared to pre-operative PWO. Conversely, Th2 polarization capacity and IL-13 production were reduced in T cells from post-operative PWO despite no detectable impairment pre-operatively, consistent with divergent innate and adaptive immune profiles after surgery.

**Conclusion:**

Type 2 immune cells showed no detectable loss of functional plasticity in human obesity despite reduced frequency. PWO after bariatric surgery show differentially reconstituted immune function, with greater macrophage plasticity and a trend towards reduced Th2 polarization capacity, despite higher GATA3 protein levels compared to PWO without bariatric surgery, arguing against uniform immune normalisation after weight loss.

## Introduction

Obesity has reached epidemic proportions worldwide, with more than one billion individuals affected in 2022 (1). The excessive accumulation of adipose tissue poses a major risk factor for many life-threatening comorbidities including type 2 diabetes mellitus, cardiovascular disease, certain types of cancer, arterial hypertension, and sleep apnoea (1, 2). The health burden of obesity challenges healthcare systems globally, necessitating a deeper understanding of the underlying pathophysiological mechanisms that link excess adiposity to metabolic and systemic disease.

White adipose tissue (WAT) has been recognized not merely as an inert energy storage depot but as a dynamic endocrine and immunological organ (3). WAT harbours various resident immune cell populations that play crucial roles in maintaining tissue homeostasis and metabolic health. These immune cells constitute a substantial fraction of the stromal vascular compartment and engage in complex bidirectional communication with adipocytes (4).

In lean healthy persons, adipose tissue maintains an anti-inflammatory microenvironment characterized by type 2 immune cell populations, including group 2 innate lymphoid cells (ILC2s), eosinophils, T helper 2 (Th2) cells, and alternatively activated macrophages (AAMs) (5–8). These cells form an integrated network: ILC2s secrete IL-5 and IL-13 to recruit and sustain eosinophils, which produce IL-4 and IL-13 to polarize macrophages toward an alternatively activated phenotype (7, 8). AAM support insulin sensitivity, while Th2 cells and regulatory T cells (Tregs) reinforce the anti-inflammatory milieu through additional IL-4, IL-13, and IL-10 secretion (9, 10). The seminal study by Wu et al. demonstrated that eosinophils are the major IL-4-expressing cells in murine WAT and are required for sustaining AAMs associated with glucose homeostasis (8). Subsequent work established ILC2s as critical upstream regulators of this circuit, with IL-33-driven ILC2 activation maintaining eosinophil recruitment and AAM polarization (11, 12).

Chronic overnutrition fundamentally disrupts this immune balance. Overweight and obesity trigger adipocyte hypertrophy, cellular stress, and hypoxia, initiating a cascade of pro-inflammatory events (13, 14). Type 2 immune cell frequencies decline substantially while pro-inflammatory populations, including classically activated macrophages (CAMs), CD8^+^ T cells, and Th1 cells, accumulate in adipose tissue (15–18). The landmark studies by Weisberg et al. and Xu et al. demonstrated that macrophages accumulate dramatically in obese adipose tissue, increasing from approx. 10% of the stromal-vascular cells in lean up to 50% in obese individuals, and represent the primary source of pro-inflammatory cytokines (13, 14). Nishimura et al. subsequently showed that CD8^+^ effector T cell infiltration into obese WAT precedes macrophage accumulation, establishing T cells as early drivers of adipose inflammation (18).

This phenotypic shift from homeostatic type 2 toward pro-inflammatory type 1/3 immunity establishes chronic low-grade inflammation that contributes to insulin resistance and metabolic dysfunction (19, 20). Importantly, this inflammatory remodelling extends beyond adipose tissue to affect immune cell populations systemically (21).

Within this framework, the PD-L1:PD-1 checkpoint has emerged as a critical regulator of adipose tissue immune homeostasis. We previously demonstrated that PD-L1 on dendritic cells limits T cell-mediated adipose tissue inflammation and ameliorates diet-induced obesity, establishing that immune checkpoints can actively maintain the type 2 environment in lean adipose tissue (22). Whether PD-1 expression on T cell subsets in human adipose tissue inflammation reflects active immune regulation rather than exhaustion during obesity remains an important question.

Bariatric surgery, including sleeve gastrectomy and gastric bypass surgery, induces substantial and durable weight loss accompanied by remarkable metabolic improvements that often precede significant weight reduction (23). Emerging evidence indicates profound immune system effects following surgery, including reduced T cell counts, decreased Th1/Th2 ratios, increased regulatory B cells, and improved NK cell activity (24–26). However, critical questions remain: Does surgery fully restore the type 2 immune landscape of lean adipose tissue? Do systemic changes parallel local adipose remodelling? Recent murine studies by Cottam et al. reveal that adipose immune cells retain obesity-associated phenotypes despite weight loss with weight regain further exacerbating inflammatory signatures (27). The concept of an “immunological scar” - persistent reprogramming of innate immunity triggered by past obesity - has been compellingly demonstrated by Hata et al. and Hinte et al., raising the question, whether human bariatric patients achieve complete immune reconstitution or exhibit persistent inflammatory imprints (28, 29).

To address these gaps, we analysed immune cell populations and functional responses in visceral adipose tissue and peripheral blood from individuals with obesity before and after bariatric surgery, and compared them to lean controls. We focussed on type 2 immune compartments - eosinophils, Th2 cells, and AAMs - testing the hypothesis that obesity depletes these populations without irreversibly impairing their functional capacity. Using flow cytometry, gene expression profiling, and *in vitro* polarization assays, we characterised both cell frequencies and functional potential. Our findings indicate that despite immune cell redistribution during obesity, type 2 immune cells show evidence of retained functional responses to appropriate stimuli *in vitro*. Cross-sectional comparison of pre- and post-operative PWO suggests partial but heterogeneous normalisation of immune profiles after bariatric surgery, with divergent trajectories for innate and adaptive immunity.

## Results

### Obesity remodels the immune cell landscape in visceral adipose tissue

To investigate the impact of obesity on resident immune cell populations, we performed flow cytometric analysis of the stromal-vascular fraction (SVF) from visceral WAT biopsies obtained during surgery from non-obese surgical controls (n=8) and people living with obesity (PWO; n=12). Clinical characteristics of our cohort are summarised in **Supplementary Table 1**. Visceral WAT was analysed in non-obese surgical controls and PWO (**Figure 1**); all subsequent analyses were performed on peripheral blood (**Figures 2**–**5**). Comparisons between pre- and post-operative PWO are cross-sectional and, unless stated otherwise, derived from different individuals.

**Figure 1:**
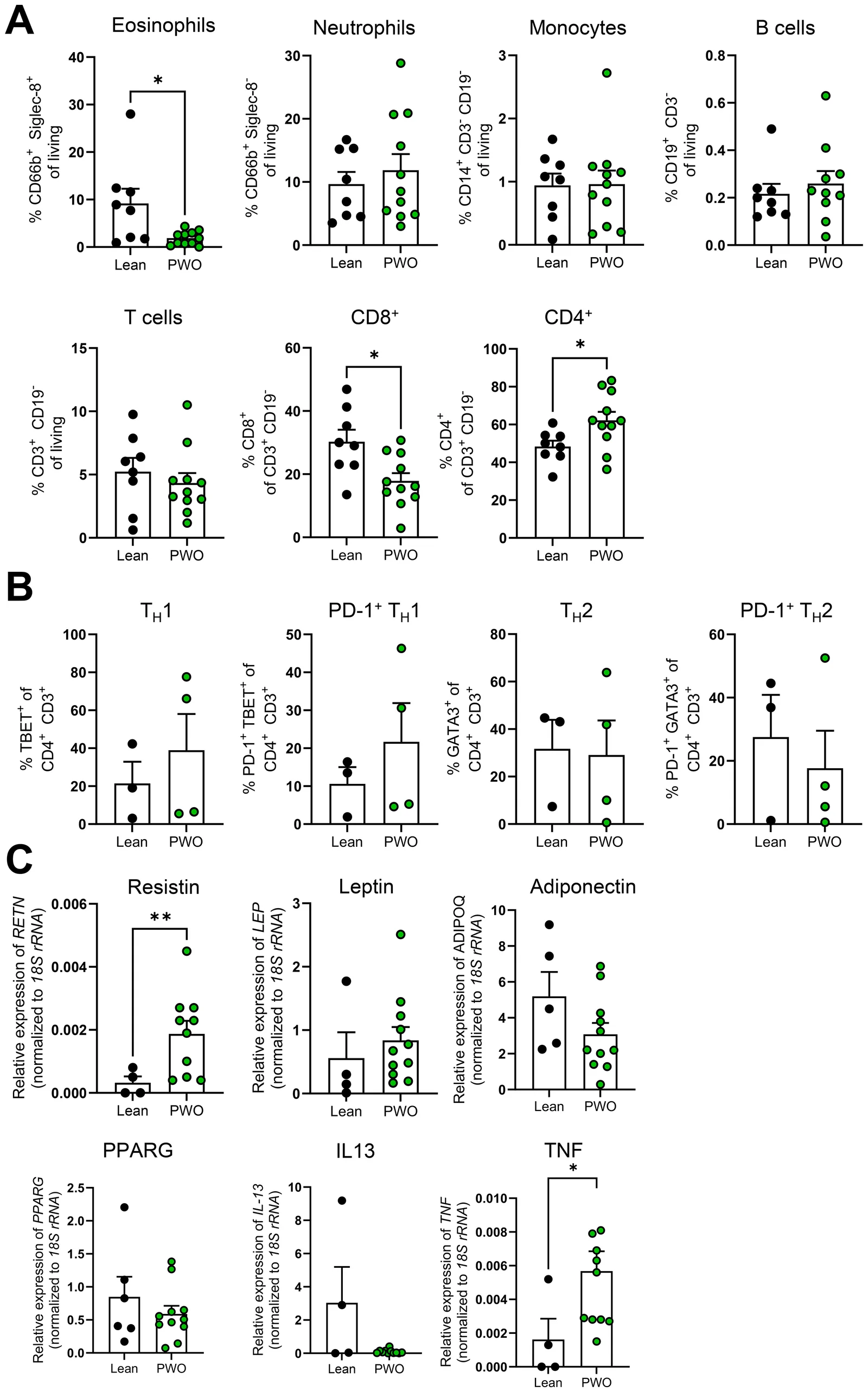
Obesity alters the immune cell composition within visceral adipose tissue. Visceral white adipose tissue (WAT) biopsies were obtained during surgery from non-obese surgical controls (n=8) and people living with obesity (PWO; n=12). **(A, B)** Flow cytometric analysis was performed on the stromal-vascular fraction (SVF) of the WAT biopsies. **(C)** Using qRT-PCR, the gene expression of Resistin (*RETN*), Leptin (*LEP*), Adiponectin (*ADIPOQ*), Peroxisome proliferator-activated receptor gamma (*PPARG*), Interleukin-13 (*IL13*) and Tumor Necrosis Factor (*TNF*) was analyzed. Relative expression was calculated using the 2^-ΔCt^ method, with human *18S rRNA* as a housekeeping gene. Exact group sizes per panel: eosinophils [Lean n = 8/PWO n = 11], neutrophils [8/11], monocytes [8/11], B cells [8/11], T cells [8/11], CD8^+^ [8/11], CD4^+^ [8/11] for A; Th1 [3/4], PD-1^+^ Th1 [3/4], Th2 [3/4], PD-1^+^ Th2 [3/4] for B; RETN [4/10], LEP [4/11], ADIPOQ [5/11], PPARG [6/11], IL13 [4/11], TNF [4/11] for **(C)** Exact p-values: eosinophils p = 0.026; CD8^+^ p = 0.018; CD4^+^ p = 0.021; RETN p = 0.006; TNF p = 0.0256. Mean + SEM. Mann-Whitney U-test.

**Figure 2:**
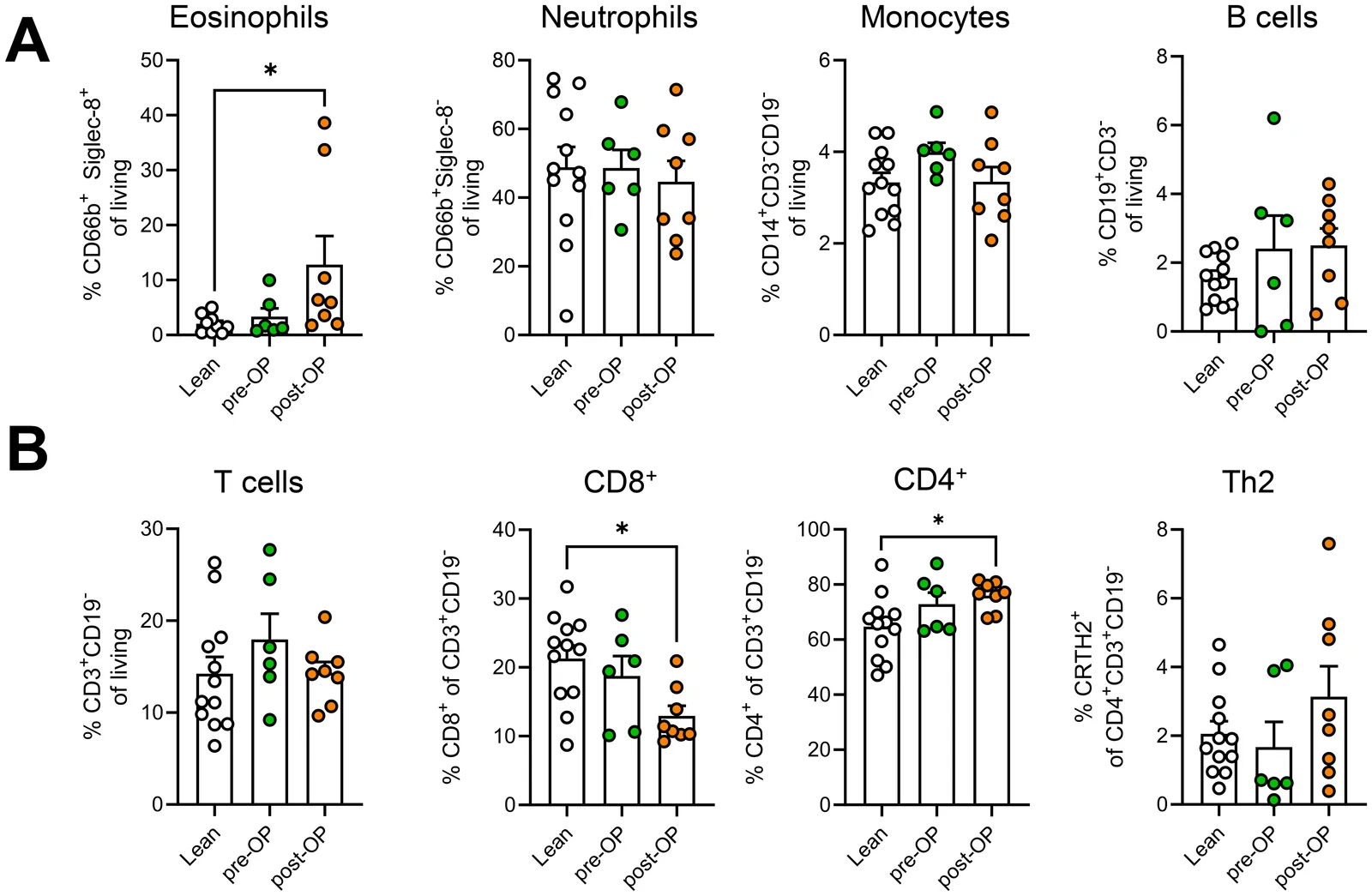
Circulating immune cell populations in obesity and after bariatric surgery. **(A, B)** Flow cytometric analysis of blood immune cells from anti-coagulated blood after depletion of erythrocytes with dextran and ACK lysis. Samples from lean controls (n=12) were compared to blood samples from PWO collected before (pre-OP; n=6) and from PWO that underwent bariatric surgery up to two years before (post-OP; n=8; median post-operative interval for this subgroup = 249 days, range 117–726 days). Exact group sizes per panel: eosinophils [lean = 12/pre-OP n = 6/post-OP n = 8], neutrophils [12/6/8], monocytes [12/6/8], B cells [12/6/8], T cells [12/6/8], CD8+ [12/6/8], CD4+ [12/6/8], Th2 [12/6/8]. Mean+SEM. Kruskal-Wallis with Dunn’s *post-hoc* test.

**Figure 3:**
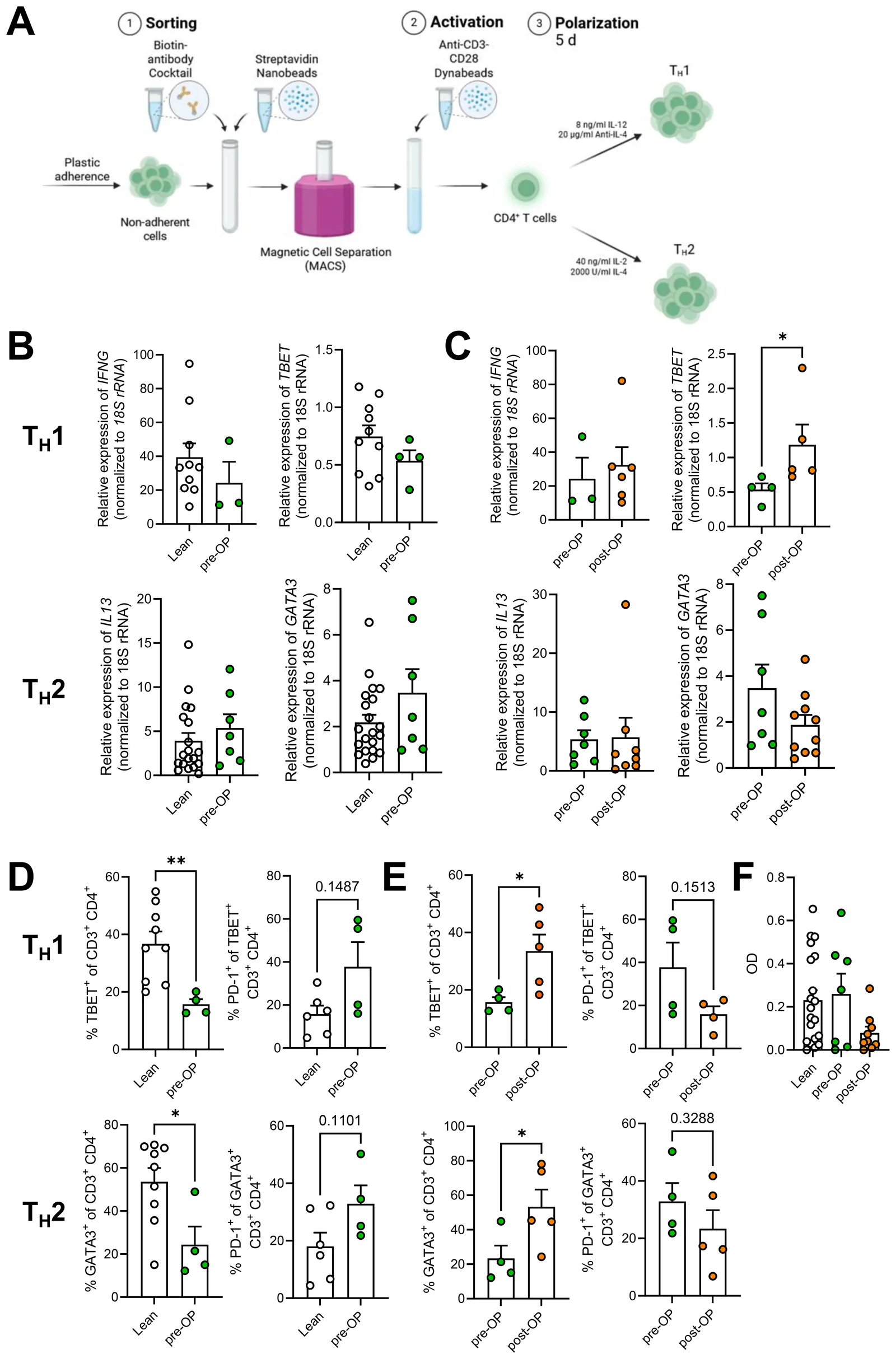
T cell polarization *in vitro*. **(A)** Schematic depiction of CD4^+^ T cell isolation from blood and *in vitro* stimulation. Unstimulated cells cultured with IL-2 alone were included as a control. Created in BioRender. Schwartz, C. (2026) https://BioRender.com/w1sb1gv. **(B, C)** Expression of Interferon-γ (*IFNG*) and T-box transcription factor 21 (*TBET*) transcripts in Th1-polarized cells and *IL13* and GATA binding protein 3 (*GATA3*) in Th2-polarized cells. Graphs show the relative expression of genes (2^-ΔCt^) normalised to *18S rRNA*. B) Polarized cells from non-obese donors were compared to PWO before surgery (pre-OP). C) Pre-OP T cells were compared to cells derived from PWO after (post-OP) bariatric surgery. **(D, E)** Flow cytometric analysis was performed from polarized cells. Relative protein expression from lean controls, pre- and postoperative patients are displayed. **(F)** IL-13 secretion by Th2-polarized T cells was quantified by ELISA. Optical density (OD) was measured at 450 nm and compared between lean, pre- and post-operative samples. Post-operative samples in this figure were obtained at a median of 476 days after surgery (range 117–2450 days). Exact group sizes per readout: IFNG [lean = 10/pre-OP n = 3/post-OP n = 6], TBET [10/4/5], IL13 [19/7/8], GATA3 [21/7/10] for B and C; TBET protein [9/4/5], PD-1^+^ TBET [6/4/4], GATA3 protein [9/4/5], PD-1^+^ GATA3 [6/4/5] for D and E; IL-13 ELISA [20/7/9] for **(F)** Mean + SEM. **, p < 0.01; *, p<0.05; Mann-Whitney U-test **(B–E)** or Kruskal-Wallis with Dunn’s *post-hoc* test **(F)**. Note that pre-operative PWO data are displayed in both panels as the shared reference group for each comparison. A combined display of all three groups (lean controls, pre-operative and post-operative PWO) for the readouts shown in panels B-E is provided in **Supplementary Figure 4**.

**Figure 4:**
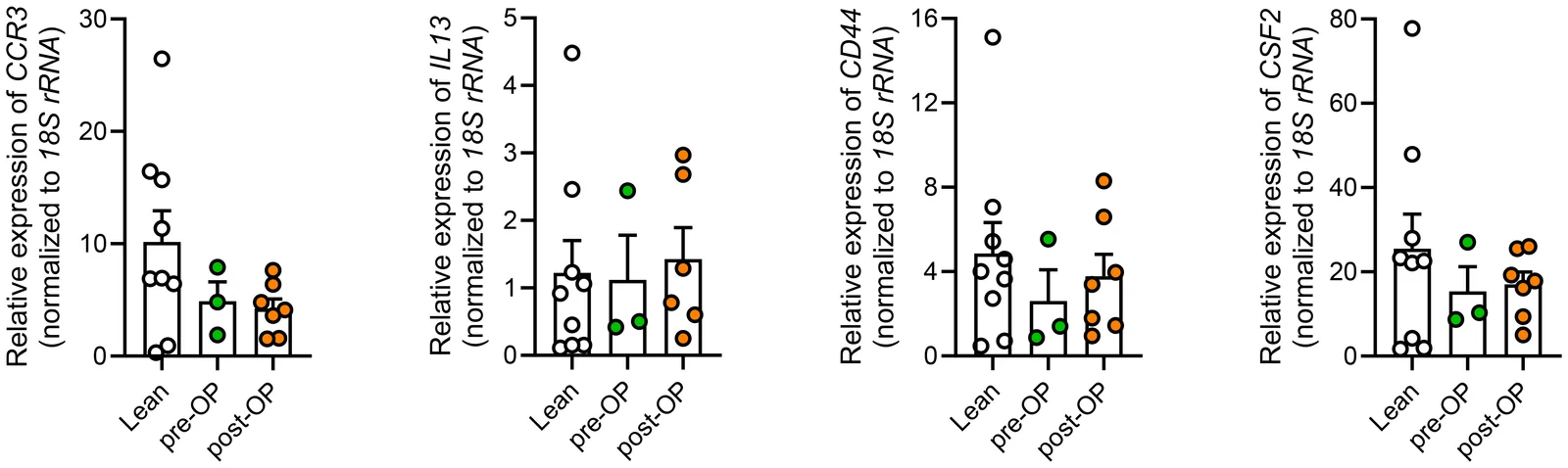
No detectable impairment of eosinophil IL-5 responsiveness in PWO. Eosinophils were isolated from peripheral blood and stimulated with IL-5 for 24 hours. Transcript expression of C-C chemokine receptor type 3 (*CCR3*), *IL13*, Cluster of differentiation 44 (*CD44*) and Colony stimulating factor 2 (*CSF2*). Expression levels were compared between lean controls (n = 9) and PWO before (pre-OP; n = 3) and after (post-OP; n = 7) bariatric surgery. Post-operative samples were obtained at a median of 208 days after surgery (range 117–334 days). Exact group sizes per transcript: CCR3 [9/3/7], IL13 [8/3/6], CD44 [9/3/7], CSF2 [9/3/7]. Two statistical outliers were identified and excluded using the ROUT method (Q = 1%) in GraphPad Prism. Eosinophils require IL-5 for survival ex vivo; unstimulated 24-hour controls were therefore not included (see Methods). Mean + SEM. Kruskal-Wallis with Dunn’s *post-hoc* test.

**Figure 5:**
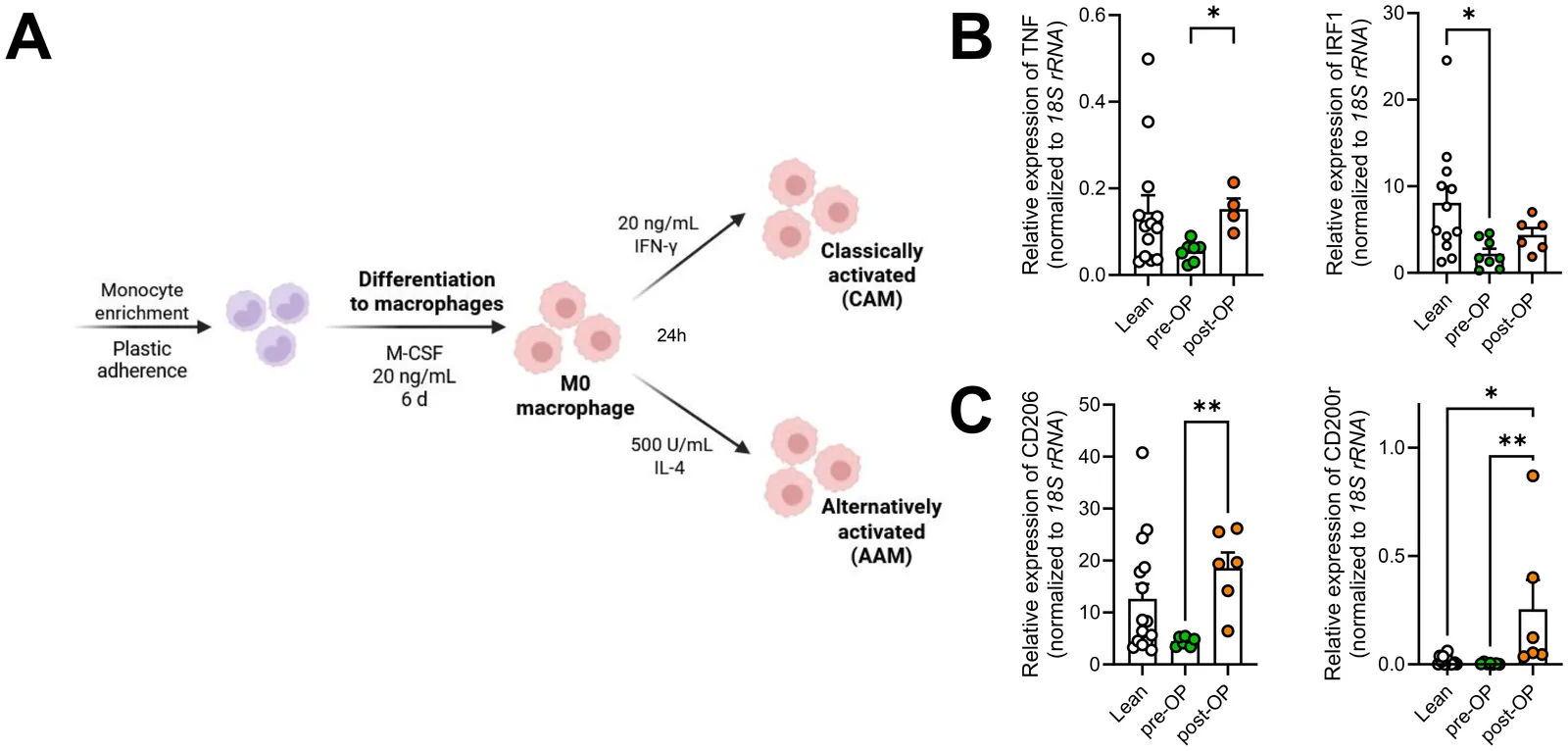
Monocyte-derived macrophages show increased polarization capacity towards AAM in PWO after bariatric surgery. **(A)** Schematic depiction of monocyte (isolated from peripheral blood) differentiation into macrophages and their subsequent polarization into classically activated macrophages (CAM) or alternatively activated macrophages (AAM). Created in BioRender. Schwartz, C. (2026) https://BioRender.com/hu3t8d2. **(B)** Gene expression in CAM-polarized cells was quantified by qRT-PCR and compared among lean controls, preoperative, and postoperative samples from PWO. Relative expression of TNF and interferon regulatory factor 1 (IRF1) was normalised to *18S rRNA*. **(C)** Gene expression in AAM-polarized cells was quantified by qRT-PCR and compared among lean controls, preoperative, and postoperative samples from PWO. Relative expression of mannose receptor C-type 1 (*CD206*) and CD200 receptor 1 (*CD200R1*) was normalised to *18S rRNA*. Post-operative samples were obtained at a median of 220 days after surgery (range 117–2118 days). Fold-change induction over non-polarizing conditions is shown in **Supplementary Figure 6**. Exact group sizes per transcript: TNF [13/7/4], IRF1 [12/8/6], CD206 [16/8/7], CD200R [16/8/7]. Statistical outliers were identified and excluded using the ROUT method (Q = 1%) in GraphPad Prism. Mean + SEM. **, p < 0.01; *, p<0.05; Kruskal-Wallis-Test with Dunn’s *post-hoc* test.

Analysis of innate immune populations revealed that eosinophil (CD66b^+^Siglec-8^+^) frequencies were significantly (p = 0.026) reduced in WAT of PWO compared to non-obese surgical controls, consistent with published murine and human data demonstrating eosinophil depletion in obese adipose tissue (**Figure 1A**) (8, 30). Neutrophil (CD66b^+^Siglec-8^-^) and B cell (CD3^-^CD19^+^) frequencies showed a small trend toward elevation in PWO but did not reach statistical significance (p = 0.5 and p = 0.5, respectively). Monocyte (CD14^+^CD3^-^CD19^-^) frequencies were comparable between groups, with considerable interindividual variability (p = 0.945).

Within the adaptive immune compartment, CD8^+^ T cells were significantly decreased in WAT from PWO (p = 0.018), while CD4^+^ T cells showed a significant increase relative to non-obese surgical controls (p = 0.021) (**Figure 1A**). Detailed T cell subset analysis revealed a shift consistent with inflammatory polarization during obesity (**Figure 1B**). Th1 cells, identified by TBET expression, were elevated in WAT from 2 out of 4 PWO compared to non-obese surgical controls. Notably, PD-1 expression on both Th1 and Th2 cells was detectable, with PD-1^+^ Th1 and PD-1^+^ Th2 populations showing trends toward differential regulation in obesity.

We further confirmed the metabolic and inflammatory remodelling associated with obesity by transcript analysis of whole WAT (**Figure 1C**). Resistin and leptin expression were elevated in PWO, consistent with adipocyte hypertrophy and metabolic dysfunction. Adiponectin, a marker of healthy adipose function, trended lower in PWO. Peroxisome proliferator-activated receptor γ (PPARγ) expression, a key transcriptional regulator of adipogenesis and adipose tissue macrophage differentiation, showed a trend toward reduction in obesity. IL-13, a canonical type 2 cytokine, was modestly reduced in PWO, while TNF expression at tissue level was significantly elevated (p = 0.0256), indicating the shift from an anti-inflammatory to a pro-inflammatory tissue milieu.

### Circulating immune cell populations reflect systemic inflammation in obesity and appear partially normalised in PWO after bariatric surgery

We next examined whether immune alterations in obesity extend to circulating immune cells and assessed the impact of bariatric surgery on immune reconstitution. Flow cytometric analysis of peripheral blood from lean controls, pre-operative PWO, and post-operative PWO revealed distinct differences across groups (**Figure 2A**).

Blood eosinophil frequencies showed variability across groups. While there was no significant difference (p > 0.99) between lean controls and pre-surgical PWO, we observed a significant elevation in PWO that underwent bariatric surgery (p = 0.025) compared to lean controls. Neutrophil frequencies showed a high degree of interindividual variability. Monocytes showed a trend toward increased frequencies in pre-operative PWO, which returned to levels of lean controls post-surgery. Circulating B cell frequencies did not differ significantly between groups (**Figure 2A**).

Within the T cell compartment, total T cell frequencies were comparable across groups, but important subset-specific changes emerged (**Figure 2B**). Circulating CD8^+^ T cell frequency was comparable between lean controls and pre-operative PWO, but were significantly (p = 0.017) decreased in PWO with bariatric surgery compared to lean controls. Conversely, CD4^+^ T cells were increased in PWO after surgery (p = 0.044) compared to lean controls. Interestingly, Th2 cells, identified by CRTH2 expression, showed a trend toward elevation in post-surgical samples. For eosinophils as well as CD8^+^ and CD4^+^ T cells, no significant difference was detected between lean controls and pre-operative PWO. Differences were only apparent between lean controls and post-operative PWO. Because pre- and post-operative samples were obtained from different individuals, we cannot formally infer causality due to surgery within individuals.

Paired analysis of pre- and post-operative blood samples was feasible for a limited subset of samples (**Supplementary Figure 2**, n=2). Despite this small sample size, these preliminary longitudinal data revealed a consistent decrease in neutrophil frequencies in both individuals following surgery-induced weight loss. However, changes in eosinophils, T cells and B cells varied considerably between the two donors, suggesting that the trajectory of immune cell reconstitution after bariatric surgery may be highly individual and likely depends on factors such as the degree of weight loss, metabolic improvement, and baseline immune status. While these paired observations are insufficient for statistical analysis, they provide initial longitudinal support for the cross-sectional differences observed in our larger cohort.

### T cells from PWO show Th2 polarization capacity comparable to non-obese controls

Having observed altered T cell subset distribution in obesity, we investigated whether functional polarization capacity was affected. We isolated naive CD4^+^ T cells from the non-adherent cellular fraction of peripheral blood, activated them with anti-CD3/CD28 beads and IL-2, and cultured cells in Th1- or Th2-polarizing conditions for five days (**Figure 3A**).

To directly address the question of whether obesity affects polarization capacity and whether bariatric surgery is associated with changes in functional responses, we performed planned pairwise comparisons to answer two biologically relevant questions. First, we compared lean and pre-operative PWO to assess the impact of obesity on polarization capacity. Second, we compared pre-operative and post-operative PWO to evaluate immune differences associated with surgical weight loss. Pre-operative data serve as the shared reference group in both comparisons.

Gene expression analysis of polarized T cells revealed robust functional responses in all groups; with the exception of *TBET*, no statistically significant differences detected between them (**Figures 3B, C**). Th1 polarization induced marked upregulation of IFN-γ mRNA in cells isolated from lean, pre-operative and post-operative donors, with no significant difference between lean and pre-OP PWO as well as pre-operative and post-operative PWO (**Figure 3B**). *TBET* expression similarly increased following Th1 polarization, confirming intact Th1 programming. Interestingly, in post-operative PWO *TBET* expression was increased compared to the pre-operative PWO cohort (**Figure 3C**, p = 0.024). Critically, Th2 polarization was not detectably impaired. IL-13 mRNA upon IL-4 stimulation was comparable between lean, pre-operative, and post-operative PWO cells (p= 0.334 and p= 0.397, respectively). GATA3, the master transcription factor for Th2 differentiation, showed comparable induction in PWO-derived T cells (**Figures 3B, C**). From the available data set we calculated fold-change of stimulated versus unstimulated samples from within each cohort and compared how well stimulation induced type 1 or type 2 associated transcripts (**Supplementary Figure 3**). Post-operative samples showed a trend towards reduction in Th2 polarization, indicating that surgical weight loss may reduce this functional property.

Flow cytometric analysis showed lower expression of TBET (p = 0.001) and GATA3 (p = 0.029) in pre-operative PWO compared to lean controls (**Figure 3D**). However, when we compared the pre-operative PWO to the post-operative cohort, we observed significantly increased expression of both TBET (p = 0.0339) and GATA3 (p= 0.0488) in PWO who already underwent bariatric surgery (**Figure 3E**). Expression of PD-1 was assessed as a marker for activation and/or exhaustion (**Figures 3D, E**). Here, we observed trends towards higher PD-1 expression on both TBET^+^ and GATA3^+^ T cells from pre-operative PWO compared to lean controls (**Figure 3D**; p = 0.1487 and p = 0.1101, respectively), and a modestly lower expression in post-operative PWO (**Figure 3E**; p = 0.1513 and p = 0.3288). In order to assess functionality of polarized T cell subsets, we measured cytokine production by ELISA (**Figure 3F**). IL-13 secretion from Th2-polarized cells did not differ significantly between lean controls and pre-OP PWO (p > 0.99). In post-operative samples, however, IL-13 secretion was non-significantly reduced (p = 0.19 vs. lean; p = 0.52 vs. pre-OP) and the fold-change induction of Th2-associated transcripts was lower (**Supplementary Figure 3**), indicating that the higher GATA3 protein levels in this group were not accompanied by a corresponding increase in type 2 effector output. In order to test whether the reduced transcription factor expression in pre-operative PWO was accompanied by a general defect in proliferation, we labelled CD4^+^ T cells with CellTrace Violet in a subset of donors (**Supplementary Figure 4**). The frequency of divided cells did not differ significantly between lean controls (n = 7) and PWO (n = 7) under non-polarizing, Th1- or Th2-polarizing conditions (p = 0.11, p = 0.14, and p = 0.14, respectively; corrected for multiple comparisons), with PWO showing a trend towards higher proliferation.

### Eosinophils from PWO show robust IL-5 responsiveness

Eosinophils play critical roles in type 2 immunity and adipose tissue homeostasis (8, 30, 31). We isolated blood eosinophils from lean controls and PWO and characterized their responses to IL-5, a key eosinophil survival and activation factor (**Figure 4**).

Gene expression analysis demonstrated that eosinophils from PWO maintained robust responses to IL-5 stimulation. CCR3, a chemokine receptor mediating eosinophil tissue recruitment, was expressed across all groups with lower expression in IL-5 stimulated cells from PWO. IL-13 showed variable expression levels with comparable responsiveness between PWO and non-obese volunteers. CD44, an activation marker associated with tissue-resident eosinophils, and CSF2 (GM-CSF), which is expressed mainly by inflammatory eosinophils, were expressed across all groups with no significant impairment in PWO (**Figure 4**).

These findings indicate that despite systemic inflammation in obesity, we detected no significant impairment of core eosinophil functional programs, including chemokine receptor expression, type 2 cytokine production, and survival signalling, in response to IL-5-induced activation ex vivo.

### Monocyte-derived macrophage polarization capacity differs between pre- and post-operative PWO

Adipose tissue macrophages are key mediators of obesity-induced inflammation. To determine whether circulating monocytes from PWO have altered polarization capacity, we generated monocyte-derived macrophages *in vitro* and exposed them to CAM-polarizing (LPS+IFN-γ) or AAM-polarizing (IL-4) stimuli (**Figure 5A**).

Unexpectedly, IFN-g-stimulation led to a trend towards increasedn TNF transcript levels in macrophages from lean controls compared to pre-operative PWO (**Figure 5B**). Macrophages derived from PWO after bariatric surgery demonstrated increased responsiveness toward IFN-γ stimulation (p = 0.0334). Similarly, IRF1, a key IFN-γ signalling mediator, showed upregulation following bariatric surgery.

Analysis of the canonical AAM marker CD206 (mannose receptor) demonstrated that IL-4 induced substantial upregulation in macrophages from lean controls (**Figure 5C**). Macrophages derived from pre-operative PWO showed decreased capacity to respond to IL-4, with lower expression of CD206 and CD200R. In post-operative PWO, however, both AAM markers were significantly increased (CD206: p = 0.008; CD200R: p =0.004) compared to pre-operative samples.

Collectively, these results demonstrate that macrophages derived from pre-operative PWO show a trend towards decreased polarization capacity both toward CAM and AAM subsets. Importantly, post-bariatric surgery macrophages showed increased responsiveness towards cytokine stimulation.

## Discussion

This study provides a focussed analysis of type 2 immune cell frequencies and functional capacity in human obesity and following bariatric surgery. Our key finding is that despite alterations in type 2 immune cell populations in visceral adipose tissue and peripheral blood during obesity, we detected no significant impairment of the core functional machinery of Th2 cells and eosinophils, whereas monocyte-derived macrophages from pre-operative PWO showed reduced responsiveness to cytokine-mediated polarization *in vitro*. Our cross-sectional analysis suggests that bariatric surgery may partially normalise immune cell composition but cannot fully return to the immune phenotype observed in non-obese persons within the timeframe examined. Notably, although our post-operative cohort achieved substantial weight loss (mean -32 kg, -10.2 kg/m² BMI), participants remained severely obese (mean BMI 43.6 kg/m²), indicating that the immune changes we observe occur in the context of persistent, albeit reduced, adiposity rather than complete metabolic normalisation. Whether further weight reduction - through sustained dietary intervention, revision surgery, or adjunct pharmacotherapy such as GLP-1 receptor agonists - would promote additional immune normalization remains an important open question. These observations have implications for understanding obesity-associated immune dysfunction and for developing targeted immunomodulatory therapies.

### Type 2 immune circuit disruption in human adipose tissue

Our observation of reduced eosinophil frequencies in obese WAT aligns with the growing body of evidence establishing eosinophils as critical regulators of adipose tissue homeostasis. Wu et al. demonstrated that eosinophils sustain AAMs in adipose tissue and that their absence exacerbates metabolic dysfunction (8). More recently, Hernandez et al. reported significantly reduced adipose tissue eosinophil content in humans with obesity, with concurrent decreases in IL-4 expression and eotaxin family members (30). Our data extend these observations by demonstrating that eosinophil depletion occurs alongside a broader restructuring of the WAT immune landscape, including decreased CD8^+^ T cells and a shift from Th2 toward Th1 predominance.

The concurrent decrease of CD8^+^ T cells in obese WAT contrasts with the seminal finding by Nishimura et al. that CD8^+^ effector T cell infiltration precedes macrophage accumulation in murine adipose tissue (18). Our data indicate that in established human obesity, CD8^+^ T cell numbers decline while CD4^+^ T cells accumulate - a pattern that may reflect species-specific differences, the chronicity of human obesity compared to short-term murine high-fat diet models, or T cell exhaustion and apoptosis in the chronically inflamed tissue milieu. Porsche et al. recently described adipose tissue T cell exhaustion in human obesity (32), which could account for the decreased CD8^+^ compartment we observe. The elevated PD-1 expression on both Th1 and Th2 cells in our WAT data connects to our prior work demonstrating that the PD-L1/PD-1 axis on dendritic cells restrains T cell-mediated adipose inflammation (22), and supports a model in which checkpoint engagement is actively attempting to limit ongoing inflammatory damage.

### Evidence for retained Th2 and eosinophil functional capacity as a therapeutic opportunity

The central translational finding of our study is that Th2 cells and eosinophils from individuals with obesity showed no detectable impairment of functional capacity when provided with appropriate polarizing signals *in vitro*. We emphasise that, given our sample sizes, the absence of a statistically significant difference does not constitute formal proof of equivalence; the data are, however, incompatible with a severe, generalised functional defect. This challenges a deterministic view of obesity-associated immune dysfunction and supports a model in which the local microenvironment, rather than irreversible cellular reprogramming, is the primary driver of impaired type 2 responses.

For T cells, our demonstration that naive CD4^+^ cells from pre-operative PWO undergo Th2 polarization with comparable IL-13 and GATA3 induction is notable. The metabolic milieu in obese adipose tissue is characterised by high leptin, low adiponectin, elevated free fatty acids, and IFN-γ dominance and may suppress Th2 differentiation through mTORC1 activation and inhibition of GATA3 transcription (20, 33). Winer et al. demonstrated that adoptive transfer of CD4^+^ T cells with intact Th2 capacity can reverse insulin resistance in obese mice (10). Our data suggest that this approach may be feasible in humans, as we detected no impairment of Th2 differentiation in cells from PWO. Intriguingly, post-operative T cells showed reduced Th2 functional output, with lower IL-13 secretion and reduced fold-change induction of Th2-associated transcripts, despite higher GATA3 protein levels in polarized cells. This dissociation between transcription factor expression and effector cytokine output suggests that the reduction in Th2 functional capacity after surgery occurs downstream of GATA3 induction rather than through a failure to upregulate the master transcription factor itself. Whether this reflects altered chromatin accessibility at the type 2 cytokine locus, changes in post-transcriptional regulation, or metabolic constraints on cytokine synthesis cannot be resolved from our data and warrants direct investigation. Proliferation of CD4^+^ T cells was not detectably reduced in PWO (**Supplementary Figure 4**), indicating that this dissociation is unlikely to reflect a general defect in T cell expansion.

For eosinophils, IL-5 responsiveness with comparable CCR3, IL-13, and CSF2 expression across groups indicates that the eosinophil activation programme was not detectably compromised. Calco et al. have emphasised that eosinophil function, rather than number alone, is the critical determinant of metabolic outcomes (31). Therapeutic strategies to increase eosinophil numbers in the WAT of PWO may therefore merit further investigation given that *ex vivo* eosinophils from PWO responded to IL-5 comparably to those from non-obese donors.

### Macrophages show increased polarization capacity after bariatric surgery

In contrast to the functional capacity of Th2 cells and eosinophils, monocyte-derived macrophages from pre-operative PWO showed a reduction in polarization capacity toward both CAM and AAM phenotypes. This functional dampening - with reduced IRF1 induction upon IFN-γ stimulation - is unexpected in light of the trained immunity framework, in which metabolic signals such as oxidised LDL and saturated fatty acids are thought to enhance innate immune responsiveness through epigenetic reprogramming (34, 35). While our study did not directly interrogate epigenetic marks, chromatin accessibility, or metabolic reprogramming in these cells, the observed phenotype is reminiscent of a tolerogenic state rather than the enhanced inflammatory priming classically attributed to obesity. We therefore hypothesise that chronic exposure of monocytes to inflammatory mediators in established, long-standing obesity may drive macrophages past the priming window into a state of hyporesponsiveness analogous to endotoxin tolerance (36, 37). In support of this concept, though not a direct test of it, Caslin et al. demonstrated that palmitic acid and conditioned medium from obese adipose tissue induce TLR4-dependent trained immunity in bone marrow-derived macrophages, an effect that was abolished by methyltransferase inhibition (38). The discrepancy between trained immunity induced by acute lipid exposure and the functional dampening we observe in monocyte-derived macrophages from PWO with long-standing obesity is consistent with the idea that duration and chronicity of metabolic stress may be a critical variable determining whether innate immune cells are primed or tolerised – a hypothesis that could be tested in future studies through assessment of histone modifications, methylation patterns, and cellular metabolic profiles in monocytes from individuals across a spectrum of obesity duration. An alternative, non-mutually exclusive explanation is that our *in vitro* differentiation protocol, which removes monocytes from their inflammatory milieu for six days of M-CSF-driven maturation, may unmask an intrinsic functional deficit that is partially compensated *in vivo* by the abundant inflammatory signals present in obese tissue.

Importantly, macrophages derived from post-operative PWO showed increased cytokine responsiveness, with TNF induction upon IFN-γ stimulation and CD206 upregulation upon IL-4 exposure comparable to macrophages from lean controls. This indicates recovery of macrophage plasticity following weight loss and suggests that the obesity-associated dampening of macrophage function may be at least partially reversible and could be driven by circulating factors, such as elevated leptin, insulin, or free fatty acids, that decrease following bariatric surgery. The differential trajectory of macrophages compared to T cells in post-surgical samples (improved macrophage polarization versus reduced Th2 capacity) highlights the distinct mechanisms governing innate versus adaptive immune reconstitution and argues against a uniform model of post-surgical immune normalisation.

### Partial and heterogeneous normalisation of immune profiles after bariatric surgery

PWO after bariatric surgery had a peripheral immune cell composition comparable to lean controls in our cohort, with notable changes in CD8^+^ and CD4^+^ T cell frequencies. These findings are consistent with CyTOF analysis of morbidly obese following surgery, who reported that naïve T cells did not fully recover within 9–11 months after surgery (24), and with Wijngaarden et al., who found that T cell differentiation profiles and cytokine-producing capacity remained altered despite B cell normalisation (26). The substantial interindividual variability in paired analyses underscores that immune reconstitution following bariatric surgery is heterogeneous and likely depends on the degree of weight loss, metabolic improvement, duration of prior obesity, and baseline immune status.

The concept of an “immunological scar” from obesity has gained support from recent studies. Hata et al. demonstrated that diet-induced obesity triggers persistent epigenetic reprogramming of the innate immune system through stearic acid and TLR4 signalling (28), and Hinte et al. showed that human adipose tissue retains transcriptional and epigenetic alterations even years after bariatric surgery (29). Most recently, the adipose niche at single-nucleus resolution across lean, obese, and weight-loss states was mapped and the study found that senescence in adipocyte progenitors and vascular cells is potently reversed by weight loss, whereas other cellular and molecular alterations persist (39). Cottam et al. used CITE-seq to demonstrate that obesity-induced imprinting of adipose immune cells persists through weight loss, with impaired recovery of Tregs and ILC2s (27). Our data add a nuanced perspective: while the immunological scar may alter macrophage responsiveness and tissue immune cell composition, the intrinsic Th2 and eosinophil differentiation potential does not appear to be permanently abolished, though formal equivalence testing was beyond the scope of this study. This distinction has therapeutic implications: interventions that shift the local microenvironment toward type 2-favouring conditions may be sufficient to re-engage the functional capacity that our data suggest is retained in these cells, without necessarily requiring reversal of epigenetic changes.

A further consideration is that bariatric surgery profoundly alters gut microbial composition and bile acid metabolism, both of which can shape systemic immune responses (40). As we did not assess the microbiome in this cohort, we cannot exclude that microbiota-driven signals contribute to the immune differences observed between pre- and post-operative PWO.

### Limitations

Several limitations warrant consideration. Lean WAT controls were obtained from patients undergoing elective abdominal surgery rather than healthy volunteers, representing the standard approach given that obtaining WAT from truly healthy individuals is ethically not feasible (30). The age difference between lean controls (mean 30.6 years) and PWO (mean 48.0 years) is a potential confounder, though age-related immunosenescence would bias against our key finding of functioning Th2 capacity (41). Most importantly, our study is predominantly cross-sectional and post-surgical immune trajectories should be read accordingly. Paired pre- and post-operative analyses were feasible in only two donors, and the post-operative sampling interval varied substantially (median 334 days, range 117–2450 days). Due to the varying follow-up times, we were unable to stratify immune phenotypes according to time post-surgery as well as to surgical procedure (sleeve gastrectomy, bypass). Our sample sizes preclude formal equivalence testing (e.g., two one-sided tests, TOST). Consequently, statements that a given function was “not impaired” should be read as the absence of a statistically detectable difference at the available power, not as demonstrated equivalence. In line with the exploratory, hypothesis-generating design of this study, we report nominal p-values without adjustment for multiple comparisons; results should be interpreted accordingly and require confirmation in adequately powered cohorts. *In vitro* polarization assays assess maximum differentiation capacity under supraphysiological conditions and may not fully recapitulate the *in vivo* milieu. Finally, we did not assess the gut microbiome, systemic metabolite profiles, or adipose tissue histology, in particular with regard to macrophage polarization, all of which change substantially after bariatric surgery and may contribute to the immune differences observed.

## Conclusions

Our data support a model, in which obesity creates a quantitative deficit in type 2 immune cells and induces macrophage hyporesponsiveness without abolishing the intrinsic functional potential of Th2 cells and eosinophils. Inferred from our cross-sectional results, bariatric surgery may differentially restore immune function through normalisation of macrophage plasticity, while paradoxically trending towards reduced Th2 capacity. These preliminary observations warrant validation in larger cohorts. Future strategies to boost type 2 immunity, such as IL-33 administration, eotaxin pathway modulation, or GLP-1 receptor agonists with emerging immunomodulatory properties (42), represent promising avenues for complementing the metabolic benefits of bariatric surgery. The retained functional responsiveness of type 2 immune cells observed here provides a rational starting point for such approaches, pending confirmation in larger, prospectively sampled cohorts.

## Methods

### Study design and participants

#### Ethics declaration

This study was conducted in accordance with the Declaration of Helsinki and approved by the local ethics committee (ethics approval number 338_21 B). All participants provided written informed consent prior to enrolment. Participation was voluntary and without financial compensation.

#### Patient cohorts

In collaboration with the Adipositaszentrum Erlangen, we enrolled 34 patients from the clinic and 37 control individuals following provision of written informed consent (**Supplementary Figure 1**). People with obesity (PWO) were diagnosed with severe obesity (BMI ≥35 kg/m^2^, grade II, or ≥40 kg/m^2^, grade III) and were scheduled for bariatric surgery or had previously undergone bariatric surgery. Blood samples were collected from 25 PWO, while WAT was collected from 12 PWO. Surgical procedures included sleeve gastrectomy (n=21), gastric bypass (n=9), sleeve gastrectomy revision (n=2), and intragastric balloon explantation (n=2). Throughout the manuscript the following designations are used: non-obese individuals donating peripheral blood are referred to as lean controls; non-obese individuals donating visceral WAT during elective surgery as non-obese surgical controls; and PWO sampled before or after bariatric surgery as pre-operative (pre-OP) and post-operative (post-OP) PWO, respectively.

The mean age of the PWO cohort was 46.3 ± 9.7 years. Mean weight before surgery was 171.32 ± 34.6 kg (BMI 54.3 kg/m^2^) and decreased to 132.3 ± 22.5 kg (BMI 43.6 kg/m^2^) after surgery, corresponding to a mean weight reduction of 32.00 ± 14.1 kg (-10.2 kg/m^2^ BMI). The cohort exhibited expected obesity-related comorbidities, including arterial hypertension (60.0%), exertional dyspnoea or obesity-hypoventilation syndrome (32.0%), orthopaedic disorders (68.0%), and metabolic disorders, such as type 2 diabetes (40.0%) (**Supplementary Table 1**). Within the PWO cohort, donors of WAT during bariatric surgery (sleeve n=9, bypass n=2, balloon n=1) had a mean age of 44.1 ± 10.8 years and a BMI of 49.8 ± 5.7 kg/m^2^. Non-obese persons, who underwent either reflux surgery (n=6) or gastric pacemaker implantation (n=2) were used as surgical controls. The non-obese cohort had a mean age of 55.8 ± 20.0 years and a mean BMI of 24.3 ± 3.1 kg/m^2^.

For the control group, blood samples were obtained from 14 male and 15 female volunteers aged 22–61 years (mean 30.6 ± 5.9 years) with a BMI <30 kg/m² (mean 23.5 ± 2.6 kg/m^2^). Non-obese controls had no documented metabolic disorders or chronic inflammatory conditions.

For each donor, we aimed to perform the full panel of analyses; however, limited sample volumes, particularly for adipose tissue biopsies and pre-operative blood draws, precluded all assays in every individual, resulting in variable sample sizes across experiments.

### Sample collection and preparation

#### Visceral adipose tissue

Visceral adipose tissue was obtained during laparoscopic surgery, stored in Ringer´s solution at 4 °C, and processed within 24 hours. A total of 12 samples were collected from PWO, and 8 samples were obtained from non-obese controls.

Adipose tissue was weighed and divided using sterile scissors for downstream analyses. 0.1-0.2 g of tissue was snap-frozen in liquid nitrogen and stored at -80 °C for subsequent gene expression analysis via quantitative PCR. An equal amount was transferred into a 50 ml Falcon tube containing 5 ml of complete RPMI medium. The tissue was mechanically minced with scissors, and 100 µl of Collagenase D (50 mg/ml; final concentration: 1 mg/ml) was added for enzymatic digestion. Samples were incubated at 37 °C for 45 min with shaking at 200 rpm. Following digestion, the cell suspension was passed through a 100 µm cell strainer, centrifuged, and red blood cells were lysed using ammonium-chloride-potassium (ACK) lysis. The remaining cells were counted, resuspended at a concentration of 0.5 × 10^6^ cells per 100 µl, and transferred to a FACS staining plate for subsequent staining.

#### Peripheral blood samples

Blood samples were collected from PWO at two time points: the initial blood sample was collected within one year prior to surgery and designated the pre-operative sample (median 43 days before surgery; range 2–344 days). Post-operative blood samples were collected at a median of 334 days (range 117–2450 days, with 4 samples >24 months) after surgery across all post-operative samples; subgroup-specific intervals are reported in the respective figure legends. At each time point, 23.5 ml of EDTA-anticoagulated blood (S-Monovetten Kalium-EDTA, Sarstedt) was collected and processed within 24 hours. For the control group, 20 ml citrate-anticoagulated blood (S-Monovette Citrat 9NC, Sarstedt) was collected and processed within 24 hours of collection. Different anticoagulants were used due to clinical sample availability, which did not affect cell frequency or marker expression (**Supplementary Figures 5A–C**).

#### Whole blood immune cell isolation

In order to analyse blood immune cell distribution, 2 ml of anticoagulated blood was transferred to a 15 ml centrifuge tube (Corning or Sarstedt) and centrifuged (5 min, 400 × g, room temperature [RT]). After removal of plasma, the remaining pellet was resuspended in Dulbecco’s phosphate-buffered saline (DPBS; DPBS w/o Ca^2+^ and Mg^2+^, PAN Biotech) to a final volume of 5 ml. Subsequently, the cell-DPBS mixture was mixed with an equal volume of 3% dextran solution (Dextran 500, Roth) and incubated at RT for 30 minutes to sediment erythrocytes. Following two rounds of ACK lysis, cells were resuspended in 200 µl RPMI medium (Roswell Park Memorial Institute [RPMI] 1640 Medium, Gibco/Life Technologies) for subsequent flow cytometric staining.

#### Isolation of peripheral blood mononuclear cells

Peripheral blood mononuclear cells (PBMCs) were isolated from 15 ml of anticoagulated blood using density gradient centrifugation. After incubation with 3% dextran solution, the erythrocyte-depleted supernatant was diluted with DPBS to a total volume of 35 ml and carefully layered on top of 15 ml Biocoll (Bio&Sell, Feucht, Germany) in a fresh 50 ml tube. Density gradient centrifugation was performed at 1,000 × g for 15 min at RT without brake. After centrifugation, the plasma layer was discarded, and the thin white interphase containing leukocytes was carefully collected and washed twice with PBS to improve purity. Cells were counted and resuspended at 1 × 10^7^ cells per 500 µl in human dendritic cell (hDC) medium (RPMI 1640 supplemented with 1% HEPES (pH 7.3), 1% human serum albumin (hSA), 1% L-glutamine, 1% penicilllin-streptomycin) for monocyte and T cell isolation, or cryopreservation. For cryopreservation, cells were centrifuged and resuspended in 500 µl 20% human serum albumin (hSA) in a cryo-tube, then incubated for 10 min on ice. Subsequently, 500 µl of freezing medium (55% hSA, 25% Glucose 40%, 20% dimethyl sulfoxide (DMSO)) was added and stored at −80 °C.

#### Monocyte-derived macrophages

Monocytes were isolated based on their adherent behaviour. A total of 500 µl of the PBMC suspension was mixed with 1,500 µl hDC medium in 6-well plates (Thermo Scientific) and incubated for 75 min at 37 °C and 5% CO_2_. Afterwards, non-adherent cells were removed and placed on ice for subsequent T cell isolation. Each well was washed three times with 2 ml RPMI. To induce differentiation into macrophages, adherent monocytes were cultured in 2 ml human macrophage medium (RPMI 1640 supplemented with 1% HEPES (pH 7.3), 10% hSA, 1% L-glutamine, 1% penicillin-streptomycin) supplemented with 20 ng/ml macrophage colony-stimulating factor (M-CSF) for 6 days (37 °C, 5% CO_2_). On day 3, 500 µl of fresh M-CSF-containing medium was added to each well.

Following the 6-day incubation period, during which monocytes differentiated into macrophages, cells were detached from the plate, washed, counted and seeded into 96-well flat-bottom plates (Thermo Scientific) at a concentration of 2 × 10^6^/ml. Cells were either left unstimulated (control), or stimulated with interferon-γ (IFN-γ, 20 ng/ml) or interleukin-4 (IL-4, 500 U/ml). Unstimulated M0 macrophages (M-CSF-differentiated, no further cytokine) were included in every experiment and served as the within-donor baseline for fold-change calculation of polarization marker induction (**Supplementary Figure 6**). After 24h, supernatants were collected and stored at -20 °C, and cells were harvested for gene expression analysis and flow cytometry.

#### T cell isolation and polarization

CD4^+^ T cells were magnetically isolated from the non-adherent cellular fraction using the Mojosort Human CD4 Naïve T Cell Isolation Kit (BioLegend) according to the manufacturer’s protocol.

2 × 10^5^ naïve T helper cells (>95% purity) were activated with 8 x 10^4^ Dynabeads Human T-Activator (Thermo Scientific) and 40 ng/ml recombinant human IL-2 (ImmunoTools). Cells were either left unstimulated or supplemented with 2,000 U/mL IL-4 (Miltenyi Biotec) for Th2 polarization, or 8 ng/ml IL-12 (Peprotech, Fisher Scientific, 10 µg/ml) and 10 µg/ml anti-IL-4 (BioXCell) antibodies for Th1 polarization. On day 3, half the medium was replaced with fresh IL-2-containing hDC medium. After 5 days of incubation (37 °C, 5% CO_2_), supernatants were collected and stored at -20 °C, and cells were harvested for gene expression analysis and flow cytometry staining. In a subset of experiments, naive CD4^+^ T cells were labelled with the CellTrace Violet Cell Proliferation Kit (Thermo Scientific) according to the manufacturer’s instructions prior to activation. Proliferation was determined by flow cytometry after 5 days as the frequency of CellTrace-diluted cells among live CD4^+^ T cells.

#### Eosinophil isolation

To isolate eosinophils, the EasySep Direct Human Eosinophil Isolation Kit (StemCell Technologies) was used according to the manufacturer’s protocol. The purified cells were counted and resuspended in eosinophil culture medium (RPMI 1640 supplemented with 20% fetal calf serum (FCS), 25 mM HEPES, 1% penicillin-streptomycin, 2 mM L-glutamine, 10 mM ß-mercaptoethanol, 10 mM non-essential amino acids). Isolation purity was assessed by flow cytometry (purity >95%). 10^5^ eosinophils were seeded per well and stimulated with either 20 ng/ml IL-5 (R&D Systems) or left untreated. After 24 h (37 °C, 5% CO_2_), eosinophils were harvested in 300 µl RNAprotect Cell Reagent (Qiagen) for downstream RNA analysis. For histological analysis, eosinophils were cultured in chamber slides using the same culture conditions. Because eosinophils undergo rapid spontaneous apoptosis in the absence of survival cytokines, unstimulated 24-hour cultures yield insufficient viable cells for reliable transcript quantification. Comparisons for eosinophils were therefore made between donor groups under identical IL-5-stimulated conditions rather than as stimulated-versus-unstimulated fold-change.

### Sample analysis

#### Flow cytometry

For flow cytometry analysis, 10^6^ cells were stained with Zombie aqua fixable dye (BioLegend) in PBS for 30 minutes at 4 °C. Cells were washed with PBS buffer. Surface staining was performed using antibodies listed in **Supplementary Table 2** diluted in FACS buffer (PBS, 2% FCS (Sigma Aldrich) and NaN_3_) and incubated for at least 30 minutes at 4 °C.

For subsequent transcription factor staining in T cells, surface-stained cells were fixed and permeabilized using FoxP3 staining buffer kit (Thermo Scientific). After incubation for 30 min at 4 °C, cells were washed with Perm/Wash buffer diluted 1:10 in PBS. Cells were stained overnight (4 °C) with antibodies listed in **Supplementary Table 2** diluted in Perm/wash buffer. Cells were washed, filtered through a 100 µm nylon mesh (Franz Eckert GmbH), and acquired on a BD LSRFortessa flow cytometer.

#### Gene expression analysis

RNA was isolated from eosinophils using the RNeasy Mini kit (Qiagen), or from T cells and macrophages using phenol/chloroform extraction with TRIzol Reagent (Thermo Scientific). Concentration and purity were measured using a NanoDrop spectrophotometer (Thermo Scientific). For each sample, a total of up to 500 ng RNA was reverse transcribed into cDNA using the High-Capacity Reverse Transcription Kit (Thermo Scientific) according to the manufacturer’s instructions. Real-time quantitative PCR was performed on a ViiA7 Real-Time PCR System (Applied Biosystems) using the SYBR Select Mastermix kit (Applied Biosystems) according to the manufacturer’s protocol. Primer sequences are listed in **Supplementary Table 3**. 18S ribosomal RNA (*18S rRNA*) was used as housekeeping gene.

#### Enzyme-linked immunosorbent assay

To quantify secreted cytokines from stimulated T cells Human IL-13 ELISA Development Kit (Peprotech, Fisher Scientific) was used according to the manufacturer’s instructions. Optical density was measured at 450 nm (Molecular Devices Spectramax 340PC).

#### Statistical analysis

All data are presented as mean ± standard error of the mean (SEM) unless stated otherwise. Normality of data distribution was assessed with the Shapiro-Wilk test; given the small group sizes, non-parametric tests were used as the default. For comparisons between two groups, the Mann-Whitney U-test was used. For comparisons of stimulated vs. unstimulated conditions within the same donor, paired tests were used. For comparisons between 3 groups, Kruskal-Wallis with Dunn’s *post-hoc* test was used.

Statistical outliers were identified using the ROUT method (Q = 1%) in GraphPad Prism. In total, 15 of 952 data points were excluded across all analyses (2 in **Figure 4** and 13 in **Figure 5**; all due to aberrant values); a sensitivity analysis including all data points did not alter the direction or statistical significance of any reported comparison. Exact p-values are reported in the Results and **Supplementary Table 4**. Effect sizes (Hedges’ g, corrected for small sample bias) and 95% confidence intervals within the groups for the principal comparisons are provided in **Supplementary Table 4**. Sample numbers (n) are described in the respective figure legends and are provided in **Supplementary Table 4**.

This study was exploratory and hypothesis-generating in design. Accordingly, p-values are reported as nominal and were not adjusted for multiple comparisons; they should be interpreted as descriptive rather than confirmatory. Sample sizes were determined by clinical sample availability and were not powered for formal equivalence testing. All statistical analyses were performed using Prism version 9.0 (GraphPad Software, San Diego, CA). P-values < 0.05 were considered statistically significant (*, p<0.05; **, p<0.01; ***, p<0.001; ****, p<0.0001).

## Data Availability

The raw data supporting the conclusions of this article will be made available by the authors, without undue reservation.
