# Supplementary material for: Divergent innate and adaptive immune trajectories after bariatric surgery in people with severe obesity
Source: Front Immunol. 2026 Oct 29;17:1951003. doi: 10.3389/fimmu.2026.1951003 (PMC13754553; doi:10.3389/fimmu.2026.1951003)
Supplement: Supplementary file 1 [file DataSheet1.pdf]

Divergent innate and adaptive immune trajectories after bariatric surgery in people with severe obesity

Jule Gawor[1], Andrea Deinzer[1], Maria Wick[1], Christian Krautz[2], Inaya Hayek[1,#], Christian Schwartz[1,3,#,*]

[1]Mikrobiologisches Institut - Klinische Mikrobiologie, Immunologie und Hygiene, Universitätsklinikum Erlangen and Friedrich-Alexander-Universität (FAU) Erlangen-Nürnberg, D-91054 Erlangen, Germany
[2]Chirurgische Klinik, Universitätsklinikum Erlangen and Friedrich-Alexander-Universität (FAU) Erlangen-Nürnberg, D-91054 Erlangen, Germany
[3]FAU Immunomedicine (FAU I-MED), Erlangen, Germany
#I.H. and C.S. are co-senior authors

Supplemental data

Supplemental Figures 1-6

Supplemental Tables 1-4

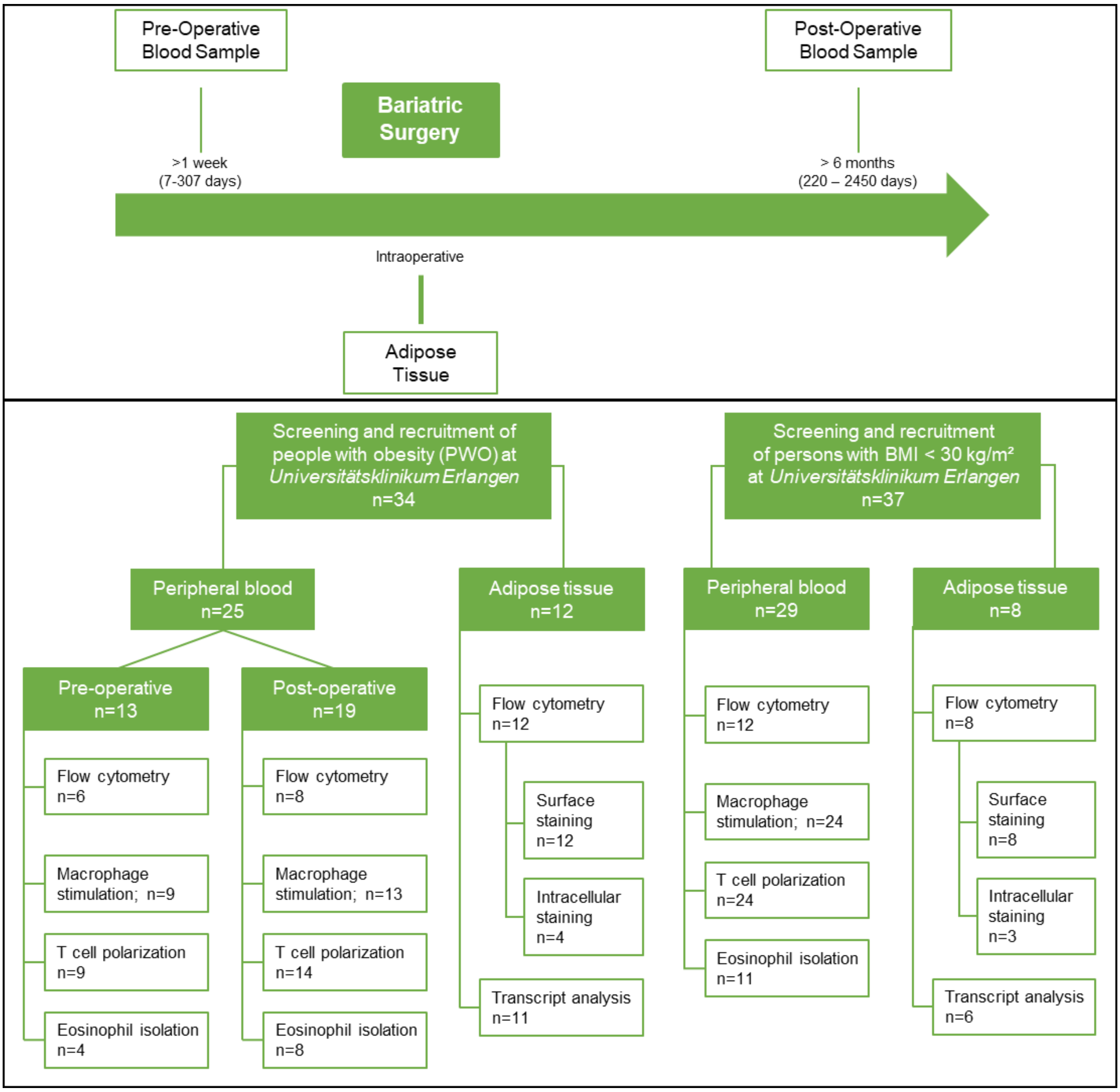

Supplemental Figure 1. Sample collection and analyses. *Upper panel*: Time line of sample collection from people with obesity (PWO). Control patient samples were collected at the same time points to match PWO analysis. *Lower panel:* Flow chart of patient samples and analyses performed from the PWO and lean cohort.

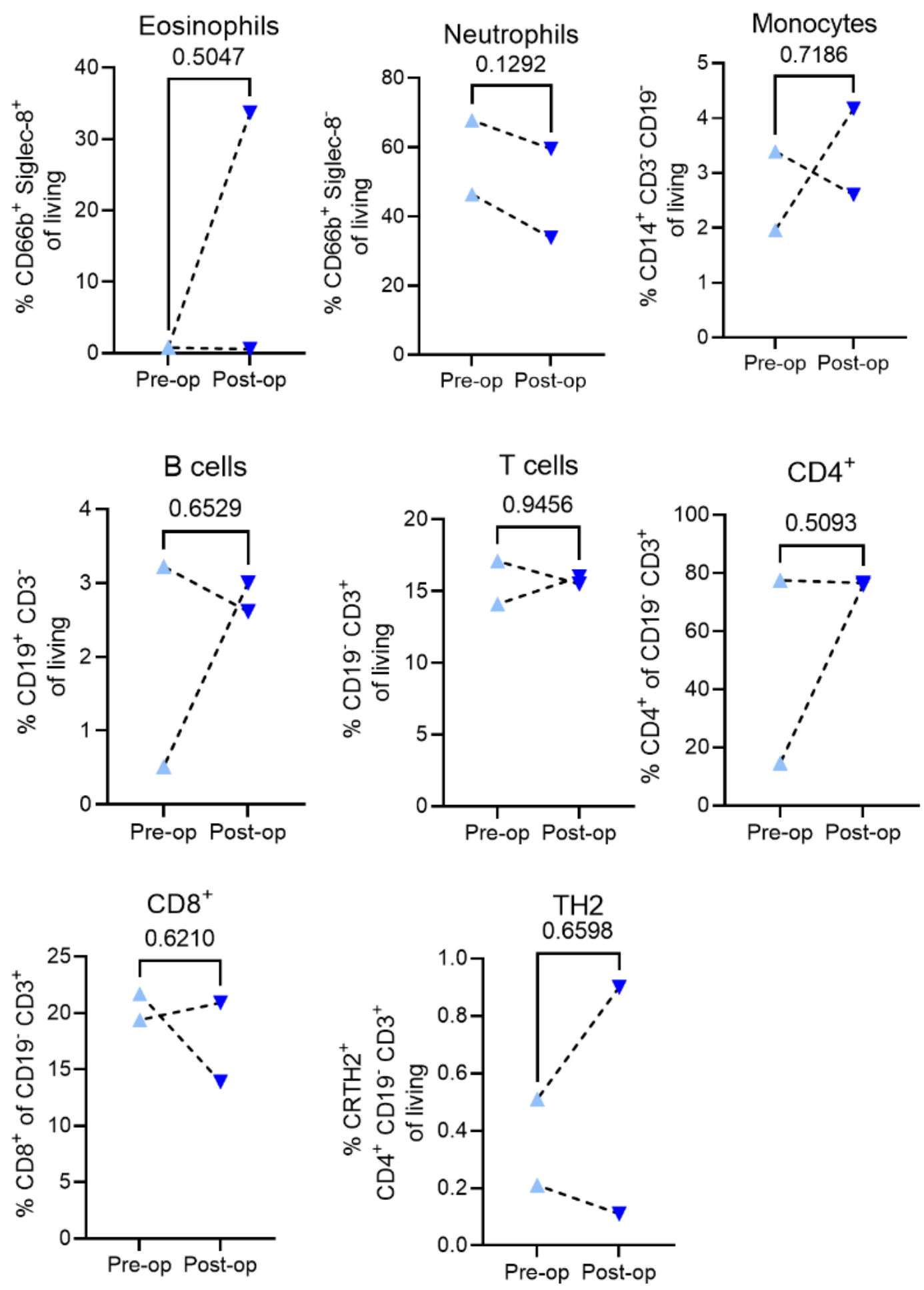

Supplemental Figure 2. Preliminary longitudinal analysis of blood samples obtained from 2 PWO patients before and after bariatric surgery. Flow cytometric analysis of blood immune cells from anti-coagulated blood after depletion of erythrocytes with dextran and ACK lysis.

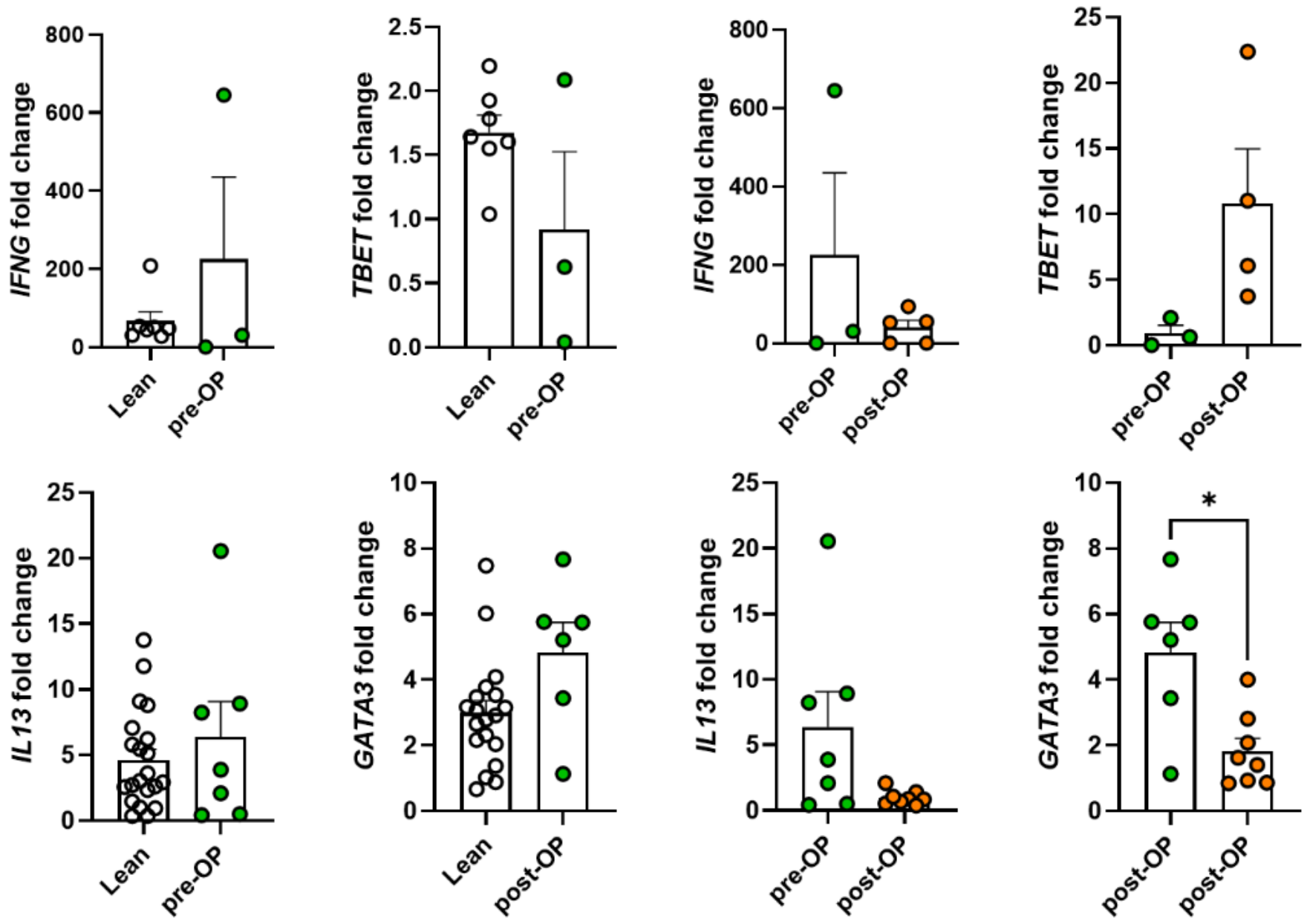

Supplemental Figure 3. Comparison of fold-change of stimulated versus unstimulated samples from within each of the lean, pre-OP and post-OP cohort to observe how well stimulation induced type 1 (upper panel) or type 2 (lower panel) associated transcripts. Expression of Interferon-γ (*IFNG*) and T-box transcription factor 21 (*TBET*) transcripts in Th1-polarized cells and IL-13 and GATA binding protein 3 (*GATA3*) in Th2-polarized cells. Mann-Whitney U-test or Welch's t test. *, $p < 0.05$.

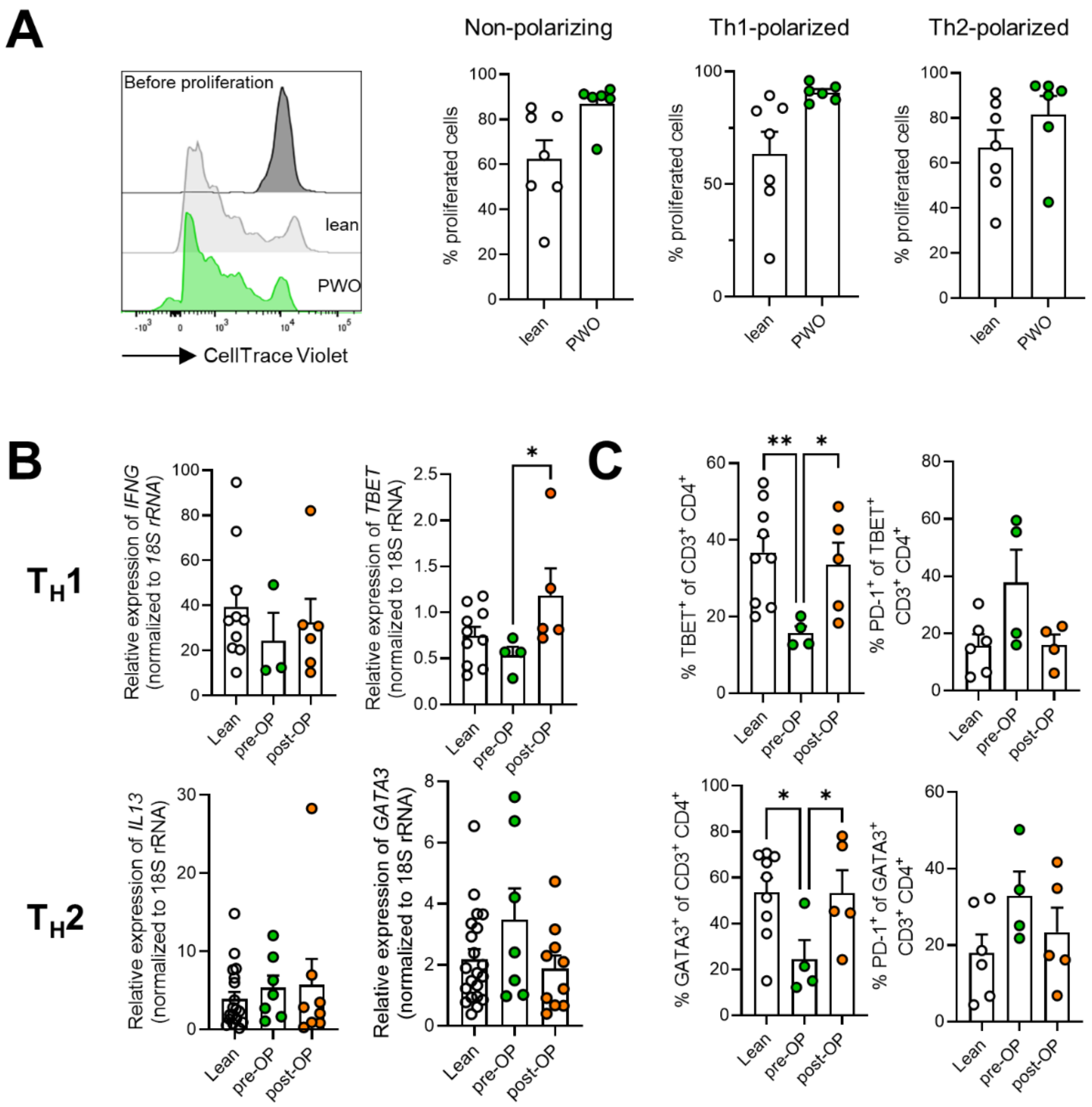

Supplemental Figure 4. CD4$^+$ T cell proliferation does not differ between lean controls and PWO. A) PBMC-derived CD4$^+$ T cells from lean controls (n = 7) and PWO (n = 7; two pre-operative, five post-operative, no matched pairs) were labelled with CellTrace Violet (left histogram of representative staining) and cultured under non-polarizing, Th1-polarizing and Th2-polarizing conditions. Shown is the frequency of divided (CellTrace-diluted) cells among live CD4$^+$ T cells; each symbol represents one donor. Mann-Whitney U test with Holm correction for three comparisons; no comparison reached significance (adjusted p = 0.11, 0.14 and 0.11 for unstimulated, Th2 and Th1 conditions, respectively). **B, C)** The data shown separately in Figure 3D and 3E are displayed here for lean controls, pre-operative and post-operative PWO in a single plot per readout, with pre-operative PWO shown once. Group sizes, statistical tests and correction for multiple comparisons are as in Figure 3D and 3E. Mann-Whitney U test.

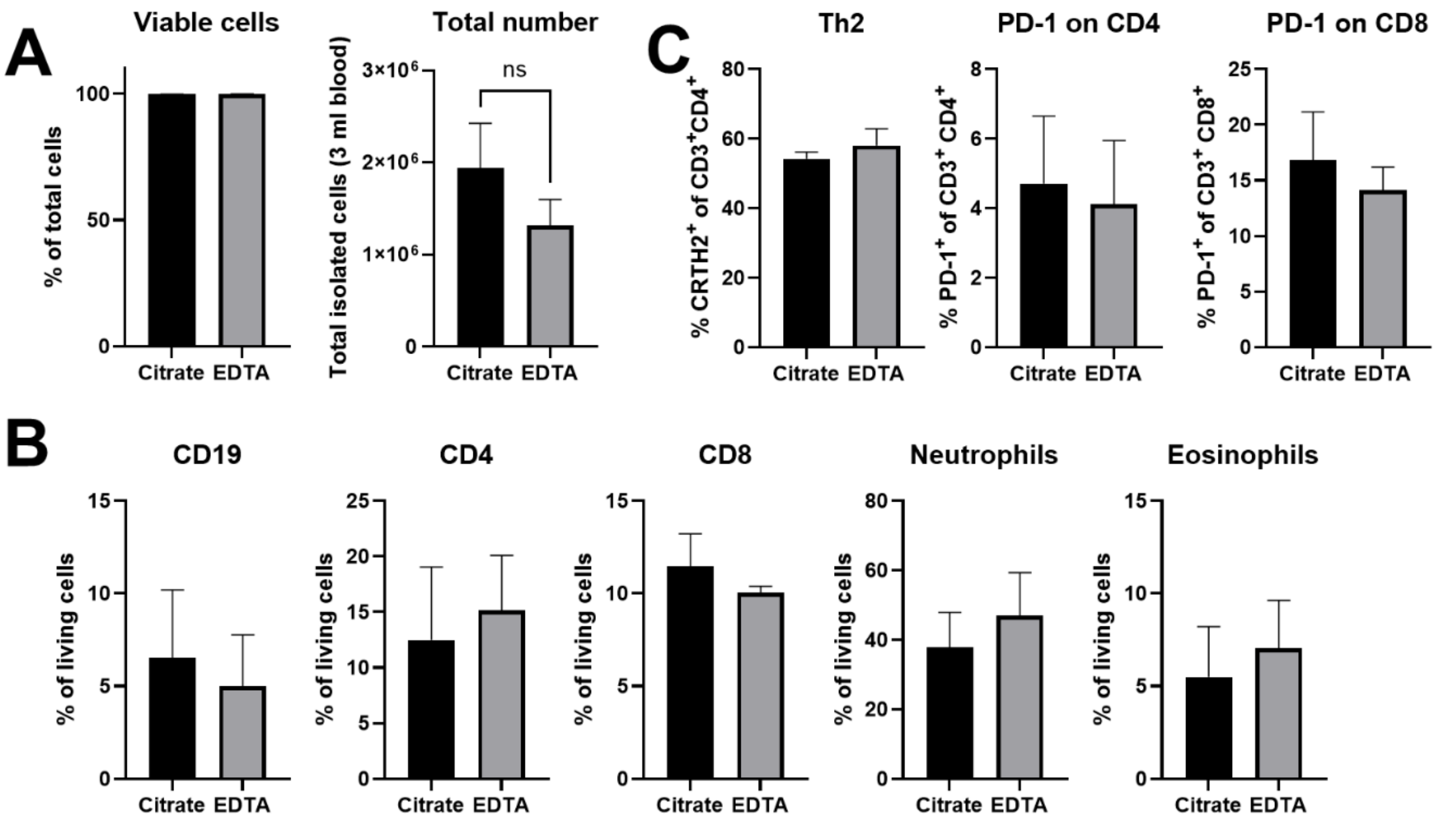

Supplemental Figure 5. Comparison of blood collected from three donors into citrate buffer (black bars) or EDTA (gray bars) collection tubes. **A)** Viable cells and total number of isolated cells from 3 ml of whole blood. **B)** Frequency of indicated cell types among living cells determined by flow cytometry. **C)** Frequency of CRTH2 on CD4 T cells and PD-1 on CD4 and CD8 T cells. n=3, ns=not significant, Wilcoxon matched-pairs signed rank test.

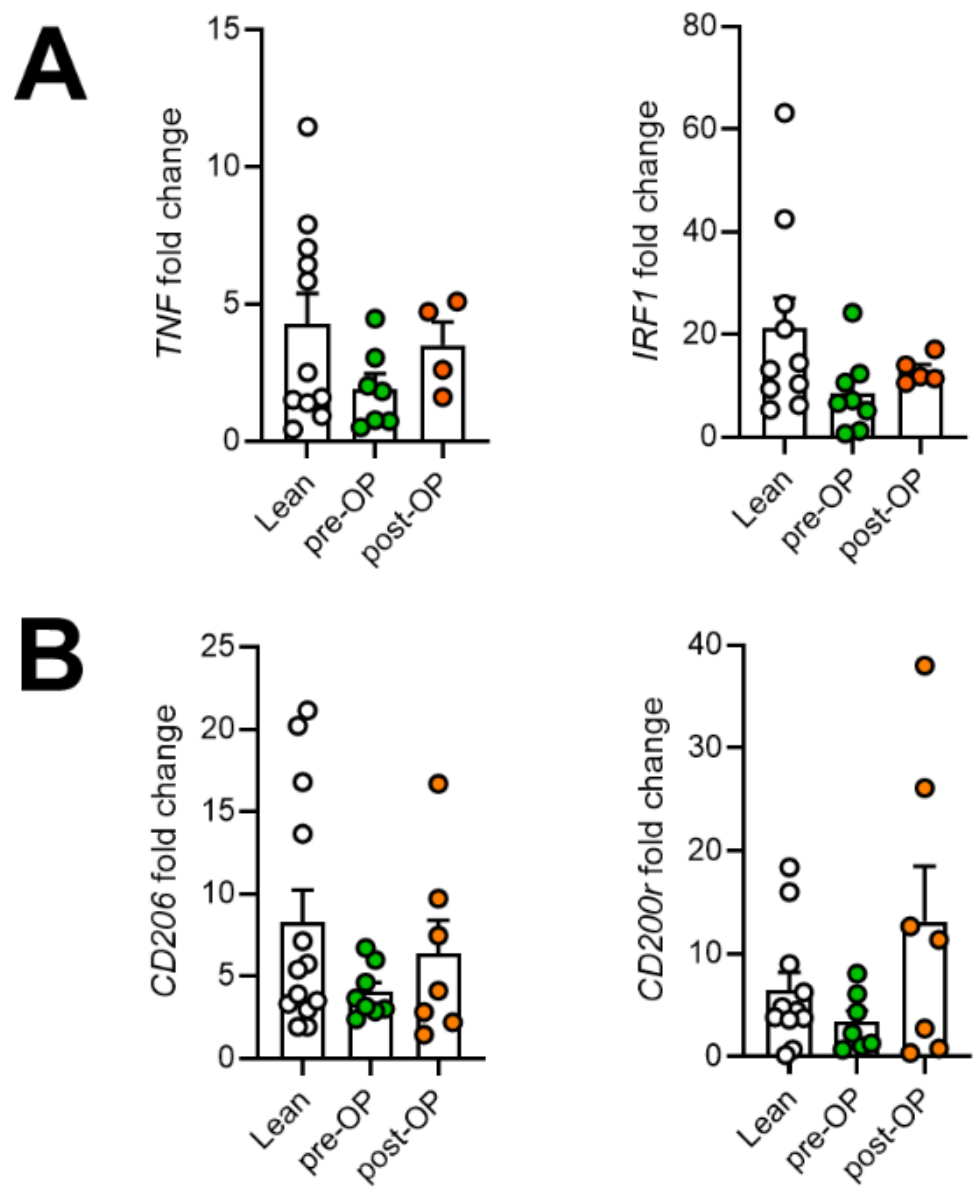

Supplemental Figure 6. Comparison of fold-change of stimulated versus unstimulated samples from within each of the lean, pre-OP and post-OP cohort to observe how well stimulation induced classically activated macrophages (CAM; upper panel) or alternatively activated macrophages (AAM; lower panel) associated transcripts. Expression of *TNF* and *IRF1* transcripts in CAM, and *CD206* and *CD200R* in AAM.

Supplemental Table 1. Demographic and clinical characteristics of study participants. Continuous variables are reported as mean ± SD. PWO, people with obesity; BMI, body mass index; GORD, gastro-oesophageal reflux disease.

	**PWO (n = 25)**	**PWO surgical (n=12)**	**Control (n = 29)**	**Lean surgical (n = 8)**
Sex (male/female)	15/10	5/7	14/15	5/3
Age (years)	46.3 ± 9.7	44.1 ± 10.8	30.6 ± 5.9	57 ± 20
Weight (kg)	150.7 ± 29.3	148.3 ± 27.2	72.1 ± 11.6	69.2 ± 9.0
BMI (kg/m²)	48.3 ± 6.9	49.8 ± 5.7	23.5 ± 2.6	24.3 ± 3.1
**Surgery type, n (%)**				
Sleeve gastrectomy	14 (56)	9 (75)	-	-
Gastric bypass	8 (32)	1 (8)	-	-
Revision	2 (8)	1 (8)	-	-
Balloon extraction	1 (4)	1 (8)	-	-
Reflux surgery	-	-	-	6 (75)
Gastric pacemaker	-	-	-	2 (25)
**Comorbidities, n (%)**				
Arterial hypertension	15 (60)	7 (58)	-	-
Type 2 diabetes mellitus	10 (40)	4 (33)	-	-
Mental disorders	10 (40)	7 (58)	-	-
Metabolic disorders	9 (36)	6 (50)	-	-
Sleep apnoea	13 (52)	5 (41)	-	-
GORD	12 (48)	4 (33)	-	-
Respiratory diseases	8 (32)	1 (8)	-	-
Orthopaedic diseases	17 (68)	7 (58)	-	-
Obesity since childhood	24 (96)	7 (58)	-	-
Genetic predisposition	19 (76)	11 (91)	-	-
	**Pre-OP (n = 13)**	**Post-OP (n = 19)**	**Mean change**	
Weight (kg)	171.3 ± 34.6	132.3 ± 22.5	- 32.0 ± 14.1	
BMI (kg/m²)	54.3 ± 6.0	43.6 ± 5.6	- 10.2 ± 4.6	

Supplemental Table 2: Antibodies used for flow cytometry.

**Antigen**	**Antibody**	**Clone**	**Source**
Blood immune cells/ WAT panel			
CD3	BUV737	OKT3	Invitrogen
CD4	PerCP-Cy5.5	OKT4	BioLegend
CD8	Brilliant Violet 785	SK1	BioLegend
CD14	BUV395	MOP9	BD Biosciences
CD19	BV421	HIB19	BioLegend
CD66b	PE/Cy7	G10F5	BioLegend
CD127 (IL-7Ra)	APC/Fire750	A019D5	BioLegend
CD161	APC	HP-3G10	BioLegend
CD294 (CRTH2)	PE/Dazzle 594	BM16	BioLegend
Siglec-8	PE	7C9	BioLegend
Lineage Cocktail 1:			
CD3	FITC	SK7	BD Biosciences
CD14	FITC	MOP9	BD Biosciences
CD16	FITC	3G8	BD Biosciences
CD19	FITC	SJ25C1	BD Biosciences
CD20	FITC	L27	BD Biosciences
CD56	FITC	NCAM16.2	BD Biosciences
T cell panel			
CD4	PerCP-Cy5.5	OKT4	BioLegend
CD3	APC/Fire 750	OKT3	BioLegend
CD25	BV711	BC96	BioLegend
PD1	PE/Cy7	EH12.2H7	BioLegend
Gata3	PE	TWAJ	Thermo Scientific
FoxP3	PE/Dazzle 594	206D	BioLegend
Tbet	APC	4B10	BioLegend
RorγT	Alexa Fluor 488, FITC	Q21-559	BD Biosciences
Macrophage panel			
CD16	PE/Dazzle 594	3G8	BioLegend
CD64	FITC	S18012C	BioLegend
CD206	BV786	15-2	BioLegend
CD274 (PD-L1)	PE/Cy7	29E.2A3	BioLegend
Eosinophil panel			
CD16	PE/Dazzle 594	3G8	BioLegend
CD66b	PE/Cy7	G10F5	BioLegend
Siglec-8	PE	7C9	BioLegend

Supplemental Table 3: Primers used for RT-qPCR.

**Target**	**Sequence 5’ → 3’**
18S rRNA	GTA ACC CGT TGA ACC CCA TT
RETN	CTG TTG GTG TCT AGC AAG ACC
LEP	TGC CTT CCA GAA ACG TGA TCC
ADIPOQ	AAC ATG CCC ATT CGC TTT ACC
PPARG	TAC TGT CGG TTT CAG AAA TGC C
IL13	CCT CAT GGC GCT TTT GTT GAC
TNF	CCT CTC TCT AAT CAG CCC TCT G
IFNG	TCG GTA ACT GAC TTG AAT GTC CA
TBET	CTG GAT GCG CCA GGA AGT TT
GATA3	GCC CCT CAT TAA GCC CAA G
CCR3	TGG CAT GTG TAA GCT CCT CTC
CD44	CTG CCG CTT TGC AGG TGT A
CSF2	AGC GGC TTC AGG ACT CTT G
IRF1	CTG TGC GAG TGT ACC GGA TG
CD206	GGG TTG CTA TCA CTC TCT ATG C
CD200R	GAG CAA TGG CAC AGT GAC TGT T

Supplemental Table 4: Descriptive statistics.

Figure	Panel	Group	Lower 95% CI	Upper 95% CI	Comparison	P-value	|Hedge's g |	n	Outliers removed
**1A**	Eosinophils	Lean	1,78	16,56	Lean vs. PWO	0,026	1,205	8	0
		PWO	1,04	3,50				11	0
	Neutrophils	Lean	5,08	14,22	Lean vs. PWO	0,500	0,286	8	0
		PWO	6,16	17,57				11	0
	Monocytes	Lean	0,50	1,38	Lean vs. PWO	0,945	0,030	8	0
		PWO	0,48	1,44				11	0
	B cells	Lean	0,13	0,22	Lean vs. PWO	0,500	0,456	8	0
		PWO	0,14	0,38				11	0
	T cells	Lean	2,69	7,78	Lean vs. PWO	0,508	0,309	8	0
		PWO	2,56	6,09				11	0
	$CD8^+$	Lean	21,24	39,26	Lean vs. PWO	0,018	1,265	8	0
		PWO	12,35	23,39				11	0
	$CD4^+$	Lean	41,19	55,61	Lean vs. PWO	0,021	1,042	8	0
		PWO	52,24	72,22				11	0
**1B**	T_H1	Lean	-27,52	70,50	Lean vs. PWO	0,472	0,456	3	0
		PWO	-22,05	99,89				4	0
	PD-1^+ T_H1	Lean	-8,50	29,68	Lean vs. PWO	0,374	0,566	3	0
		PWO	-10,75	54,13				4	0
	T_H2	Lean	-20,71	84,15	Lean vs. PWO	0,895	0,085	3	0
		PWO	-17,26	75,42				4	0
	PD-1^+ T_H2	Lean	-30,07	85,07	Lean vs. PWO	0,609	0,352	3	0
		PWO	-20,03	55,38				4	0
**1C**	*Resistin*	Lean	-0,00030	0,00095	Lean vs. PWO	0,006	1,249	4	0
		PWO	0,00093	0,00281				10	0
	Leptin	Lean	-0,74	1,86	Lean vs. PWO	0,226	0,362	4	0
		PWO	0,37	1,31				11	0
	Adiponectin	Lean	1,42	8,97	Lean vs. PWO	0,211	0,819	5	0
		PWO	1,68	4,48				11	0
	PPARG	Lean	0,07	1,63	Lean vs. PWO	0,458	0,451	6	0
		PWO	0,32	0,86				11	0
	IL13	Lean	-3,84	9,91	Lean vs. PWO	0,489	1,118	4	0
		PWO	0,01	0,17				11	0
	TNF	Lean	-0,00229	0,00554	Lean vs. PWO	0,026	1,061	4	0
		PWO	0,00308	0,00829				11	0
**2A**	Eosinophils	Lean	0,78	3,33	Lean vs. pre-OP	0,999	0,503	12	0
		pre-OP	-0,51	7,24	Lean vs. post-OP	0,025	1,109	6	0
		post-OP	0,48	25,11	pre-OP vs. post-OP	0,159	0,768	8	0
	Neutrophils	Lean	35,74	61,94	Lean vs. pre-OP	1,000	0,011	12	0
		pre-OP	35,10	62,17	Lean vs. post-OP	0,868	0,208	6	0
		post-OP	30,21	59,04	pre-OP vs. post-OP	0,912	0,241	8	0
	Monocytes	Lean	2,86	3,80	Lean vs. pre-OP	0,205	0,942	12	0
		pre-OP	3,46	4,52	Lean vs. post-OP	0,999	0,019	6	0
		post-OP	2,58	4,11	pre-OP vs. post-OP	0,270	0,785	8	0
	B cells	Lean	1,12	2,01	Lean vs. pre-OP	0,478	0,555	12	0

		pre-OP	-0,07	4,89	Lean vs. post-OP	0,339	0,873	6	0
		post-OP	1,34	3,67	pre-OP vs. post-OP	0,992	0,048	8	0
**2B**	T cells	Lean	10,20	18,27	Lean vs. pre-OP	0,411	0,543	12	0
		pre-OP	10,71	25,20	Lean vs. post-OP	0,999	0,020	6	0
		post-OP	11,58	17,11	pre-OP vs. post-OP	0,486	0,660	8	0
	$CD8^+$	Lean	17,11	25,48	Lean vs. pre-OP	0,683	0,359	12	0
		pre-OP	11,31	26,19	Lean vs. post-OP	0,017	1,388	6	0
		post-OP	9,54	16,39	pre-OP vs. post-OP	0,203	0,977	8	0
	$CD4^+$	Lean	57,43	71,90	Lean vs. pre-OP	0,232	0,702	12	0
		pre-OP	61,95	83,72	Lean vs. post-OP	0,044	1,143	6	0
		post-OP	71,58	80,40	pre-OP vs. post-OP	0,820	0,378	8	0
	T_H2	Lean	1,26	2,86	Lean vs. pre-OP	0,906	0,256	12	0
		pre-OP	-0,22	3,55	Lean vs. post-OP	0,416	0,559	6	0
		post-OP	1,04	5,23	pre-OP vs. post-OP	0,319	0,614	8	0
**3B**	T_H1 *IFNG*	Lean	20,89	57,96	Lean vs. pre-OP	0,469	0,558	10	0
		pre-OP	-29,26	77,93				3	0
	T_H1 *TBET*	Lean	0,53	0,97	Lean vs. pre-OP	0,240	0,704	10	0
		pre-OP	0,25	0,83				4	0
	T_H2 *IL13*	Lean	2,05	5,79	Lean vs. pre-OP	0,334	0,356	19	0
		pre-OP	1,58	9,16				7	0
	T_H2 *GATA3*	Lean	1,50	2,88	Lean vs. pre-OP	0,250	0,671	21	0
		pre-OP	0,96	5,99				7	0
**3C**	T_H1 *IFNG*	pre-OP	-29,26	77,93	pre-OP vs. post-OP	0,714	0,256	3	0
		post-OP	5,40	59,53				6	0
	T_H1 *TBET*	pre-OP	0,25	0,83	pre-OP vs. post-OP	0,024	1,129	4	0
		post-OP	0,37	2,00				5	0
	T_H2 *IL13*	pre-OP	1,58	9,16	pre-OP vs. post-OP	0,397	0,045	7	0
		post-OP	-2,10	13,55				8	0
	T_H2 *GATA3*	pre-OP	0,96	5,99	pre-OP vs. post-OP	0,193	0,751	7	0
		post-OP	0,89	2,86				10	0
**3D**	T_H1 % TBET+ of CD3+ CD4+	Lean	26,83	46,62	Lean vs. pre-OP	0,001	1,756	9	0
		pre-OP	10,02	21,38				4	0
	T_H1 %PD-1+ TBET+ of CD3+ CD4+	Lean	5,77	25,79	Lean vs. pre-OP	0,149	1,248	6	0
		pre-OP	1,34	74,21				4	0
	T_H2 % GATA3+ of CD3+ CD4+	Lean	38,81	68,28	Lean vs. pre-OP	0,029	1,462	9	0
		pre-OP	-2,35	51,10				4	0
	T_H2 % PD-1+ *GATA3+ of CD3+ CD4+*	Lean	5,58	30,45	Lean vs. pre-OP	0,110	1,104	6	0
		pre-OP	12,65	53,15				4	0
**3E**	T_H1 % TBET+ of CD3+ CD4+	pre-OP	10,02	21,38	pre-OP vs. post-OP	0,034	1,580	4	0
		post-OP	17,51	49,49				5	0
	T_H1 %PD-1+ TBET+ of CD3+ CD4+	pre-OP	1,34	74,21	pre-OP vs. post-OP	0,151	1,116	4	0
		post-OP	4,26	27,64				4	0
	T_H2 % GATA3+ of CD3+ CD4+	pre-OP	-0,27	47,02	pre-OP vs. post-OP	0,049	1,248	4	0

		post-OP	25,37	81,15				5	0
	T_H2 % PD-1+ *GATA3+ of CD3+ CD4+*	pre-OP	12,65	53,15	pre-OP vs. post-OP	0,329	0,478	4	0
		post-OP	-0,85	50,65				5	0
**3F**	OD	Lean	0,13	0,33	Lean vs. pre-OP	0,999	0,127	20	0
		pre-OP	0,03	0,49	Lean vs. post-OP	0,198	0,822	7	0
		post-OP	0,01	0,15	pre-OP vs. post-OP	0,521	0,969	9	0
**4**	*CCR3*	Lean	3,77	16,56	Lean vs. pre-OP	0,999	0,645	9	0
		pre-OP	-2,60	12,36	Lean vs. post-OP	0,296	0,866	3	0
		post-OP	2,13	6,35	pre-OP vs. post-OP	0,999	0,234	7	0
	IL13	Lean	0,12	2,32	Lean vs. pre-OP	0,992	0,313	8	1
		pre-OP	-1,72	3,96	Lean vs. post-OP	0,952	0,601	3	0
		post-OP	0,24	2,62	pre-OP vs. post-OP	0,940	0,241	6	1
	CD44	Lean	1,50	8,23	Lean vs. pre-OP	0,999	0,510	9	0
		pre-OP	-3,74	8,95	Lean vs. post-OP	0,999	0,272	3	0
		post-OP	1,22	6,33	pre-OP vs. post-OP	0,999	0,389	7	0
	CSF2	Lean	6,58	44,41	Lean vs. pre-OP	0,692	0,416	9	0
		pre-OP	-9,87	40,59	Lean vs. post-OP	0,639	0,416	3	0
		post-OP	9,83	24,19	pre-OP vs. post-OP	0,991	0,177	7	0
**5B**	*TNF*	Lean	0,06	0,23	Lean vs. pre-OP	0,131	0,771	13	3
		pre-OP	0,03	0,08	Lean vs. post-OP	0,779	0,049	7	1
		post-OP	0,07	0,23	pre-OP vs. post-OP	0,033	2,634	4	0
	IRF1	Lean	3,94	12,22	Lean vs. pre-OP	0,018	1,082	12	1
		pre-OP	0,81	3,60	Lean vs. post-OP	0,999	0,637	8	0
		post-OP	2,37	6,42	pre-OP vs. post-OP	0,294	1,151	6	0
**5C**	*CD206*	Lean	6,56	18,75	Lean vs. pre-OP	0,204	0,867	16	2
		pre-OP	3,71	5,27	Lean vs. post-OP	0,243	0,562	8	2
		post-OP	10,77	26,32	pre-OP vs. post-OP	0,008	2,610	7	0
	CD200R	Lean	0,01	0,08	Lean vs. pre-OP	0,942	0,677	16	2
		pre-OP	0,00	0,01	Lean vs. post-OP	0,016	0,801	8	2
		post-OP	-1,45	4,31	pre-OP vs. post-OP	0,003	0,632	7	0
